# Genome-Wide Detection of Serpentine Receptor-Like Proteins in Malaria Parasites

**DOI:** 10.1371/journal.pone.0001889

**Published:** 2008-03-26

**Authors:** Luciana Madeira, Pedro A. F. Galante, Alexandre Budu, Mauro F. Azevedo, Bettina Malnic, Célia R. S. Garcia

**Affiliations:** 1 Departamento de Parasitologia, Instituto de Ciências Biomédicas, Universidade de São Paulo, São Paulo, Brasil; 2 Departamento de Bioquímica, Instituto de Química, Universidade de São Paulo, São Paulo, Brasil; 3 Ludwig Institute for Cancer Research, São Paulo, Brasil; 4 Departamento de Fisiologia, Instituto de Biociências, Universidade de São Paulo, São Paulo, Brasil; Federal University of São Paulo, Brazil

## Abstract

Serpentine receptors comprise a large family of membrane receptors distributed over diverse organisms, such as bacteria, fungi, plants and all metazoans. However, the presence of serpentine receptors in protozoan parasites is largely unknown so far. In the present study we performed a genome-wide search for proteins containing seven transmembrane domains (7-TM) in the human malaria parasite *Plasmodium falciparum* and identified four serpentine receptor-like proteins. These proteins, denoted PfSR1, PfSR10, PfSR12 and PfSR25, show membrane topologies that resemble those exhibited by members belonging to different families of serpentine receptors. Expression of the pfsrs genes was detected by Real Time PCR in *P. falciparum* intraerythrocytic stages, indicating that they potentially code for functional proteins. We also found corresponding homologues for the PfSRs in five other *Plasmodium* species, two primate and three rodent parasites. PfSR10 and 25 are the most conserved receptors among the different species, while PfSR1 and 12 are more divergent. Interestingly, we found that PfSR10 and PfSR12 possess similarity to orphan serpentine receptors of other organisms. The identification of potential parasite membrane receptors raises a new perspective for essential aspects of malaria parasite host cell infection.

## Introduction

Malaria is the major human parasitic disease. A recent study revealed over 500 million clinical cases of *P. falciparum* malaria worldwide, a number 50% higher than that reported by the World Health Organization [1]. The control of this disease has long been a problem, despite intense research efforts, possibly because of the complex biology of *Plasmodium*, and the ability of this organism to conceal itself within host cells. The symptomatic stage of malaria infection concurs with the development of the asexual cycle of the parasites in the red blood cell [2].

Analysis of *P. falciparum* gene expression profiles revealed that a programmed cascade of cellular processes ensures completion of the parasite's intraerythrocytic developmental cycle [3], [4]. An emerging series of data are now accumulating concerning the effectors of second messengers such as Ca^2+^-activated proteases, cAMP-dependent kinases, Ca^2+^-dependent kinases and metabolic enzyme effectors [5], [6]. The question then arises: how would parasites sense the environment to activate signaling machineries responsible for the control of their growth and differentiation?

We have reported that the host hormone melatonin evokes synchronization of intraerythrocytic stages by causing Ca^2+^ release from the intracellular stores of *Plasmodium*, during the asexual cycle. The melatonin effect is specifically blocked by luzindole and U73122, which are, respectively, a melatonin receptor antagonist and a phospholipase C (PLC) inhibitor, thus suggesting a signal transduction pathway that acts through activation of a parasite membrane receptor coupled to PLC [7], [8]. By entrapping fluorescent Ca^2+^ probes selectively at the parasitophorous vacuolar space Gazarini *et al.* [8] demonstrated that the vacuolar space indeed contains high levels of Ca^2+^, which provides the necessary conditions for intracellular Ca^2+^ signaling in the malaria parasites. Melatonin signaling pathway in *Plasmodium* is well established and involves a cross-talk between the second messengers Ca^2+^ and cAMP [9]. In addition, one of the possible targets for the melatonin-elicited Ca^2+^ stimulus in malaria parasites was recently described as a Ca^2+^-dependent cysteine-protease, which activation was monitored with peptide probes and analyzed by Fluorescence Resonance Energy Transfer [10].

Another environmental factor sensed by malaria parasites is xanthurenic acid (XA), present in the mosquito, which is a key modulator of differentiation of gametocytes into male and female gametes within the mosquito midgut [11], [12]. XA triggers a rise in cytosolic Ca^2+^ in gametocytes. Bilker *et al.* [13] identified a Ca^2+^ activated kinase (CDPK4) that appears essential for the sexual reproduction and mosquito transmission in *P. berghei*.

Heptahelical or serpentine receptors mediate physiological responses to stimuli as diverse as light, odorants, bitter and sweet tastants, pheromones, hormones, neurotransmitters, small peptides, proteins, lipids and ions [14]. According to the classical view, serpentine receptors couple to downstream effectors such as adenylyl or guanylyl cyclases, phospholipases A2 or C and ion channels, via heterotrimeric guanine nucleotide-binding proteins (G-proteins). However, it is now evident that many heptahelical receptor-mediated processes function independently of G-proteins [14], [15]. Serpentine receptors comprise the most widespread class of membrane receptors, with members in fungi, plants, and all the metazoan organisms. In spite of their conserved seven-transmembrane (7-TM) architecture, heptahelic receptors make up a highly divergent family, with members within each family sharing only 25% amino acid sequence identity in the conserved transmembrane core region, while among different families little sequence similarity is shared [16].

The identification of parasite membrane receptors is a fundamental task and should shed light on essential aspects of malaria parasite biology. In this article, we report the identification of four putative heptahelical receptors in *P. falciparum*, all of them expressed during the intraerythrocytic cycle of the parasite. Homologues for these receptors in other *Plasmodium* species were also identified.

## Results

### Detection of serpentine receptor-like genes in the *P. falciparum* genome

**Figure 1:**
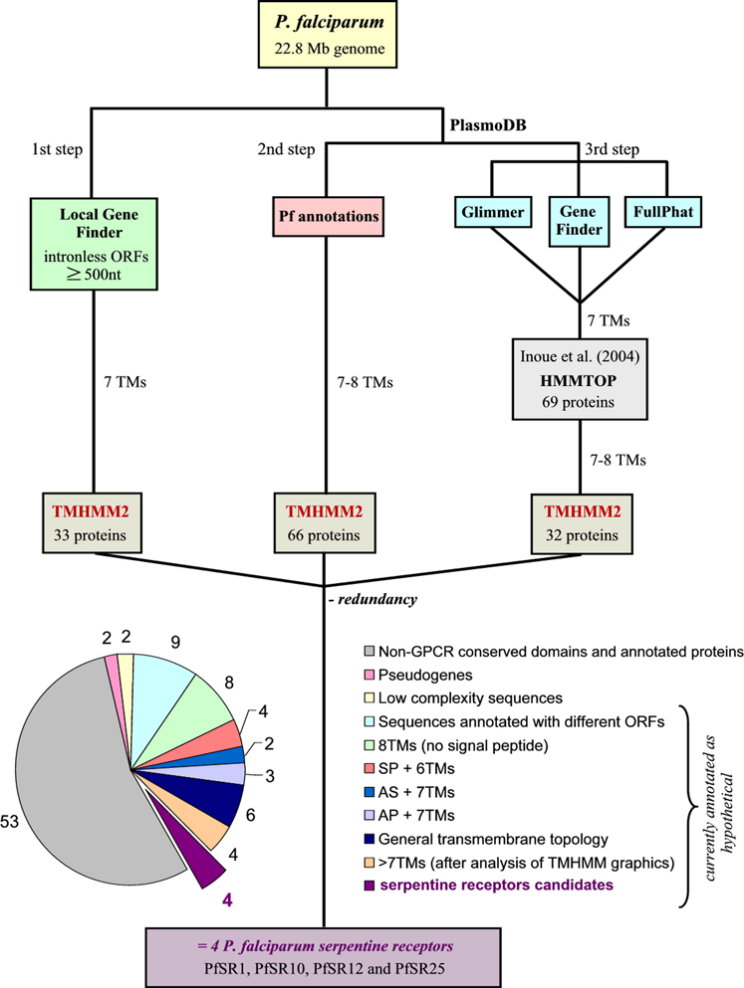
Summary of the strategy used to search the *P. falciparum* genome for serpentine receptor-like genes. The genomic sequences available from PlasmoDB (release 4.3) were analyzed in three steps. In the first step, we used a local ORF-finder program to predict intronless ORFs longer than 500 nucleotides (green box) and searched these ORFs for seven transmembrane 7-TM proteins by using the TMHMM 2.0 transmembrane prediction program. In the second step, *P. falciparum* manually annotated genes (pink box) available in PlasmoDB were analyzed using TMHMM2.0 to select 7- and 8-TM proteins. In the third step, we show the automatically predicted sequences used in the work of Inoue *et al.* [17], which were retrieved by the authors from PlasmoDB (blue boxes – Glimmer, Gene finder and FullPhat are gene-finding programs) followed by their analysis with HMMTOP transmembrane prediction program to select 7-TM proteins (gray box). As our third step we reanalyzed the 69 proteins described by Inoue *et al.* [17], now using TMHMM2.0. The redundant 7- and 8-TM proteins retrieved by the three steps were eliminated and the sequences were subsequently classified according to the criteria set listed on the bottom of the figure. The graphic on the left shows the absolute number of sequences for each criteria class during the analysis, emphasizing the four *P. falciparum* serpentine receptor-like proteins. SP, signal peptide; AS, anchor signal; AP, apicoplast signal.

We searched the *P. falciparum* genome for serpentine receptor-like genes. Members belonging to the serpentine receptor super family are highly divergent in their amino acid sequences, making it difficult to identify new members through homology searches. However, all these receptors have in common a central core domain constituted of seven transmembrane helices (TM-1 through TM-7) connected by three intracellular loops and three extra-cellular loops. Therefore, we decided to search the genome for proteins that contain seven putative transmembrane domains. A summary of the strategy we used is shown in Figure 1.

The whole procedure can be divided into three steps. The first step was to create a program to identify all of the intronless open reading frames (ORFs) present in the *P. falciparum* genome. The identified ORFs were then translated and the resulting amino acid sequences were searched for the presence of TMs using the TMHMM 2.0 prediction program. The TMHMM 2.0 program was chosen since it is considered to be a good predictor for genuine GPCRs (G-protein coupled receptors). However, we can not exclude the possibility that we have missed *Plasmodium* serpentine receptor candidates because we used TMHMM 2.0 in the initial screening. We selected the sequences containing 7-TMs and obtained thirty-three candidates. Since genes containing introns were omitted in this first analysis, in the second step we analyzed all the annotated *P. falciparum* ORFs (Pf annotations) available from PlasmoDB. The tool “Gene Sequence Features – transmembrane domains” was used to search for ORFs containing 7- and 8-TMs according to TMHMM 2.0 program, because some sequences with eight predicted TMs actually contain seven TMs plus one N-terminal peptide signal. A total of sixty-six candidates were selected in this step.

Previously, Inoue and colleagues [17] reported the identification of 46 serpentine receptors from the *P. falciparum* genome. In this case, the authors used *P. falciparum* genes that had been automatically predicted by the PlasmoDB consortium using three different gene prediction programs (Glimmer, Gene finder and FullPhat – [18]) and scanned their corresponding amino acid sequences for the presence of seven TMs using HMMTOP 2.0. As our third step, we reanalyzed these 46 sequences using TMHMM 2.0 and manually inspected their membrane topologies. Thirty-two of the receptors showed convincing 7- or 8-TM architectures, and they were added to the candidates list obtained in steps 1 and 2.

The redundant candidates were eliminated and the resulting 98 sequences were further analyzed according to a series of stringent parameters, such as conserved amino acid sequence motifs, sequence similarity with non-serpentine receptor proteins, the presence of repeats (in low-complexity sequences) and sorting signals. As shown in Figure 1, more than half of the sequences clearly have non-serpentine receptor functions, such as transporters, ATPases, and proteins involved in fatty acid and ceramide synthesis. We also eliminated sequences with 8-TMs but no N-terminal signal peptide (8%) and proteins not showing characteristic serpentine receptor transmembrane topologies according to different prediction programs. Other eliminated proteins had a signal peptide plus only 6-TMs, or a signal anchor with no cleavage site followed by a 7-TM core.

Interestingly, we found three heptahelical proteins with an apicoplast transit peptide (PFL0655w, PF14_0435 and PF14_0607), which are hypothetical proteins exclusive of the *Plasmodium* genus as evidenced by BLASTp searches. These proteins share approximately 37% of amino acid sequence similarity to each other and may constitute a novel family of *Plasmodium* apicoplast serpentine receptor-like proteins and could represent potential new targets for antimalarial drugs. However, as their sub cellular location is most likely the apicoplast, they were not further analyzed in this study.

It is important to emphasize that we were highly stringent in the last stages of transmembrane topology analysis of the *P. falciparum* proteins, eliminating all the sequences that showed extra-TMs with medium probability scores (0.4<*p*<0.7) in the TMHMM 2.0 graphics but which were not considered as a transmembrane domain in the final TMHMM model, as it is the case of PFC0650w, PFC0970w, PFF0645c, PFF0890c, PF13_0028 and PF11_0334 (represented in Figure 1 as proteins with more than 7-TMs after analysis of TMHMM graphics).

Our analysis finally resulted in the identification of four promising *P. falciparum* serpentine receptor-like proteins, denominated PfSR1, PfSR10, PfSR12 and PfSR25, all of them currently annotated as hypothetical proteins. The PfSR-like candidates and their general characteristics are summarized in Table 1. The PlasmoDB IDs and chromosome locations are shown for each one of the genes. Comparison of the gene structure with their corresponding cDNAs indicates that *pfsr12* and *pfsr25* genes contain introns, whereas *pfsr1* and *pfsr10* do not. This was confirmed for *pfsr10*, *pfsr12* and *pfsr25* by sequencing the products amplified by RT-PCR with primers matching to the 5′ and 3′-ends of the predicted ORFs (data not shown). We were, however, not able to amplify the *pfsr1* full-length ORF (2,322 bp long) from cDNA samples.

**Table 1:** *P. falciparum* serpentine receptor-like candidates.

					Transmembrane Prediction Algorithm					
*P. falciparum* serpentine receptors	PlasmoDB ID	Chr	Introns*	Number of aminoacids in the predicted protein	TMHMM2	TMAP	TOPPRED2	TMPRED	HMMTOP	SignalP
**PfSR1**	PF11_0321	11	No	773	7	8 (SP+7TM)	8 (SP+7TM)	8 (SP+7TM)	8 (SP+7TM)	No
**PfSR10**	PFL0765w	12	No*	655	8 (SP+7TM)	7 (SP+6TM)	8 (SP+7TM)	8 (SP+7TM)	8 (SP+7TM)	Yes
**PfSR12**	PFD1075w	14	6*	470	7	7 (SP+6TM)	7	6	8 (SP+7TM)	Yes
**PfSR25**	MAL7P1.64	7	1*	357	8 (SP+7TM)	7 (SP+6TM)	8 (SP+7TM)	8 (SP+7TM)	8 (SP+7TM)	Yes

The PlasmoDB identification number, chromosome location, number of introns and number of amino acids in the predicted protein are indicated for each one of the four PfSR candidates. The presence/absence of introns marked with an asterisk was confirmed by sequencing the full-length cDNAs amplified by RT-PCR using primers matching to the 5′ and 3′-ends of the predicted open reading frames (Data S1). The number of transmembrane helices predicted by five different programs is also shown for all PfSRs. The existence of potential signal peptides as predicted by SignalP program is indicated. (SP+nTM) means that N-terminal signal peptide was recognized as a transmembrane (TM) spanning segment, which was followed by “n” other TM domains.

The different *P. falciparum* serpentine receptor-like predicted proteins vary in their sizes: the largest translated ORF belongs to PfSR1 (773 amino acids) and the shortest belongs to PfSR25 (357 amino acids). It is known that different topology prediction programs vary in their performances [19]. We decided to compare the membrane topology predictions for the four different proteins using four additional programs. As shown in Table 1, the great majority of the programs predict seven TMs for all of the proteins, indicating that our initial predictions were correct.

We also used the SignalP algorithm to scan the putative receptors for the presence of signal peptides and their cleavage sites. Potential signal peptides were identified for PfSRs10, 12 and 25. Although no signal peptides were predicted by SignalP for PfSR1, other four transmembrane prediction programs pointed to the presence of an N-terminal signal peptide as shown in Table 1.

### The *P. falciparum* serpentine receptor-like proteins show variable membrane topologies

**Figure 2:**
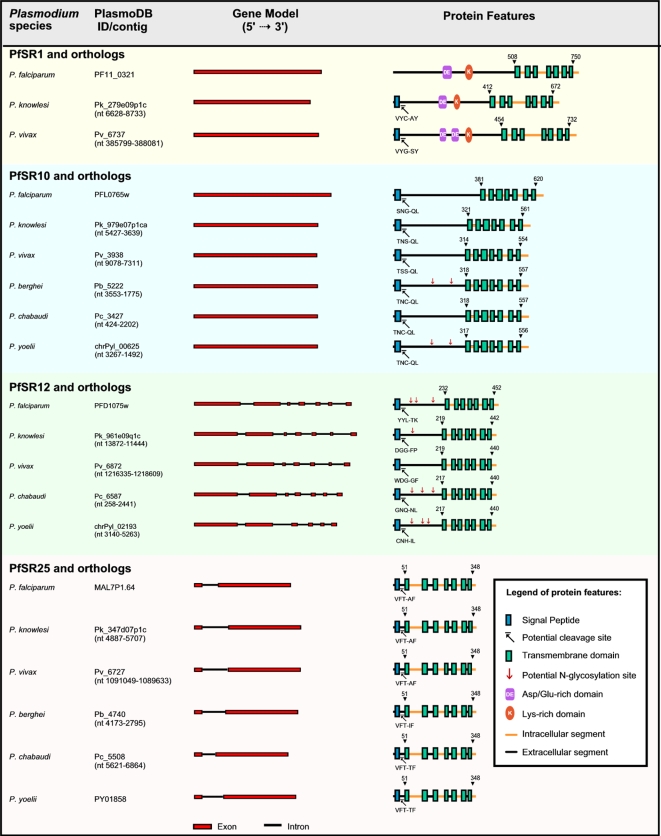
Characteristics of serpentine receptor-like proteins (SRs) of diverse malaria parasites species. Each one of the four *P. falciparum* serpentine receptors sequences are grouped with its orthologs and differently colored: yellow, PF11_0321 (PfSR1); blue, PFL0765w (PfSR10); green, PFD1075w (PfSR12); pink, MAL7P1.64 (PfSR25). Identification numbers for most of the non-falciparum sequences are not available since these genes are not annotated in the databases; therefore, we point out the contig sequences from where they were retrieved with the nucleotides that comprise the selected gene. Scale-sized diagrams of genomic structure of serpentine receptor genes (Gene Model) and of predicted protein features are shown. Transmembrane domains predicted by TMHMM2.0 are shown in dark-green boxes, with the number of amino acids in the beginning and in the end of the 7-TM core. Potential signal peptides predicted by SignalP3.0 are shown as blue boxes with the potential cleavage sites. In the N-terminal domains of some of these receptors there are repetitive stretches of negatively charged residues (Glu/Asp-rich domains) or positively charged residues (Lys-rich domains). The position of potential N-glycosylation sites is pointed by red-arrows.

Vertebrate PfSRs are divided into three main families based on sequence similarity: family A (the rhodopsin family), family B (the secretin family) and family C (the metabotropic glutamate receptor family). Usually the TM topology pattern is conserved among PfSRs that have the same function or that belong to the same family [20], [21]. Bioinformatic predictions of membrane topologies of the four PfSRs are presented in Figure 2. All proteins have the characteristic seven transmembrane helices connected through loops. The N-terminal regions are located extracellularly and the C-terminal tail is extended into the cytoplasmic side of the membrane. The lengths of the N-terminal domains are variable in the different receptors. PfSR1 possesses a very large extracellular N-terminal domain (508 amino acids long), like members of family C, which includes the glutamate metabotropic and GABA receptors. PfSR10 also has a large N-terminal domain (381 amino acids); similar sizes were described for hormone receptors such as the follicle stimulating hormone receptor (FSHR) and the luteinizing hormone receptor (LHR), which belong to family A. The other PfSRs have shorter N-terminal domains, the shortest being the one from PfSR25 (22 amino acids). Several family A members, like odorant, adenosine and adrenalin receptors, also have small N-terminal domains. The loops connecting the TMs are not very variable among the different PfSRs, except for the first intracellular loop (i1) in PfSR25, which is longer than the others (62 amino acids long, see Figure 2).

These observations are in agreement with the classification obtained by Inoue *et al.* [17] using the binary topology pattern methodology for the lengths of loops and tails of serpentine receptors, where PfSR1 was classified as a member of Class C (Glutamate Metabotropic Receptor subclass), and PfSR10 and PfSR25 as members of Class A (Hormone Receptor and Olfactory Receptor subclasses, respectively). PfSR12 was included in the Inoue *et al.* [17] analysis as the FullPhat automatic gene prediction (chr4.phat_243); in fact, this gene prediction is probably a composition of two distinct genes, a helicase (PFD1070w) and the heptahelical receptor PfSR12 (PFD1075w), which were correctly separated at the final PlasmoDB annotation.

In conclusion, in spite of the absence of amino acid sequence similarity with well-characterized serpentine receptors, the *P. falciparum* serpentine receptor-like proteins show overall 7-TM architectures that resemble the ones exhibited by members belonging to different families of serpentine receptors.

### *Plasmodium* serpentine receptor-like homologues

We next investigated the presence of serpentine receptor-like homologues in other *Plasmodium* species. The amino acid sequences of PfSRs were used to scan the genome sequences from two other primate parasites (*P. vivax and P. knowlesi*) and from three rodent parasites (*P. chabaudi, P. berghei and P. yoelii*). Most of the tBLASTn searches retrieved parasite sequences that are truncated or not annotated.

Comparative gene-finding methods have proved to be powerful tools to assign gene function for related organisms [22], [23]. In order to identify more reliable gene structures for the PfSR homologues, we performed a homology-based gene prediction using the program FGENESH+, which is one of the most accurate programs available at the moment [23] and is also trained to predict intron splice sites in *P. falciparum*. We recomputed gene predictions from genomic regions of other *Plasmodium* species containing predicted exons with significant protein similarity to the PfSRs by using FGENESH+. However, it is important to mention that in the case of determining the gene structure of *pfsr12* orthologues in other *Plasmodium* species we had to manually inspect and correct the predictions of introns/exons. Since we had amplified the full-length of *pfsr12* by RT-PCR and sequenced it, we were certain about its intron-exon gene structure; with this knowledge in conjunction with analysis using FGENESH+ and the algorithm for homology comparison ‘BLAST 2 sequences’, we were able to identify the most reliable gene predictions. Finally, we analyzed the synteny maps provided by PlasmoDB to confirm the orthology of these genes. Predicted protein and nucleotide sequences as well as alignments of *Plasmodium* serpentine receptor homologues and for phylogenetic tree building are available in Data S1, S2, S3, S4.

For Data S1 only sequences corresponding to the 7-TM core regions were used. Multiple sequences alignment was done by ClustalW version 1.8.

Table 2 shows the amino acid sequence similarities between each one of the PfSRs and its corresponding sequence in another species. Only sequences showing ≥50% of amino acid sequence similarity (ASS) were considered to be true homologues. PfSR10 and 25 have homologues in all species with similarities >70%. The PfSR1 and PfSR12 homologues show lower similarities to the *P. falciparum* sequences (ranging from 51.6 to 62.8% ASS). PfSR1 homologues were found only for the primate parasites, but not for the rodent parasites. In addition, the two primate counterparts (PvSR1 and PkSR1) are 77% similar to one another and only 52.5 and 55.7% similar to PfSR1. All these findings were confirmed with the analysis of chromosomal synteny available in PlasmoDB. These results strongly indicate that *P. falciparum* has a different and unique serpentine receptor-like protein, PfSR1, which is absent from the other species. Nevertheless, we cannot exclude the possibility that gaps in the different *Plasmodium* genome sequences have precluded us from identifying some of the homologues.

**Table 2:** Similarities among *Plasmodium* serpentine receptor-like protein homologues.

	% of aminoacid sequence similarity				
	Pv	Pb	Py	Pc	Pk
**PfSR1**	52.5	-	**41.9**	**39.7**	55.7
**PfSR10**	77.1	73.5	74.2	73.4	77.6
**PfSR12**	**46.5**	-	60.2	62.8	51.6
**PfSR25**	92.2	87.4	87.4	87.7	91.9

The amino acid sequence similarity between each one of the PfSRs and each one of its corresponding homologue in another species is shown. Proteins showing similarities bellow 50% (shown in bold) were not considered to be true homologues. No similar protein sequences were found for PfSR1 in *P. berghei*. Only a partial sequence was found for PfSR12 in *P. berghei*, and it was not included in our analysis.

**Figure 3:**
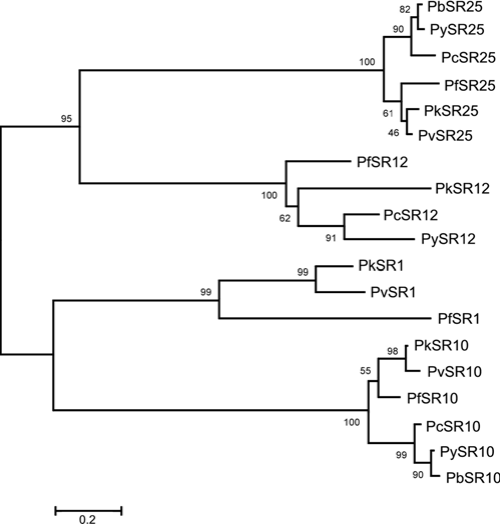
Phylogenetic tree showing the comparison of the 7-TM core of the *Plasmodium* serpentine receptors amino acid sequences. Bootstrap values are shown at nodes with >50% support. (Scale bar: 0.2 amino acid substitutions per site). The bar indicates the number of amino acids substitution per site.

To investigate the sequence relationships among the candidate receptors we generated a multiple alignment using the 7-TM core regions of all of the *Plasmodium* sequences and constructed a phylogenetic tree. As shown in Figure 3, each one of the four PfSRs, together with their corresponding homologues, constituted an individual branch. Each branch was subdivided into two smaller branches, one containing the primate parasite homologues, and the other the rodent parasite homologues. Branches corresponding to PfSRs1 and 12 show the highest divergence among the different *Plasmodium* homologues, as discussed above.

**Figure 4:**
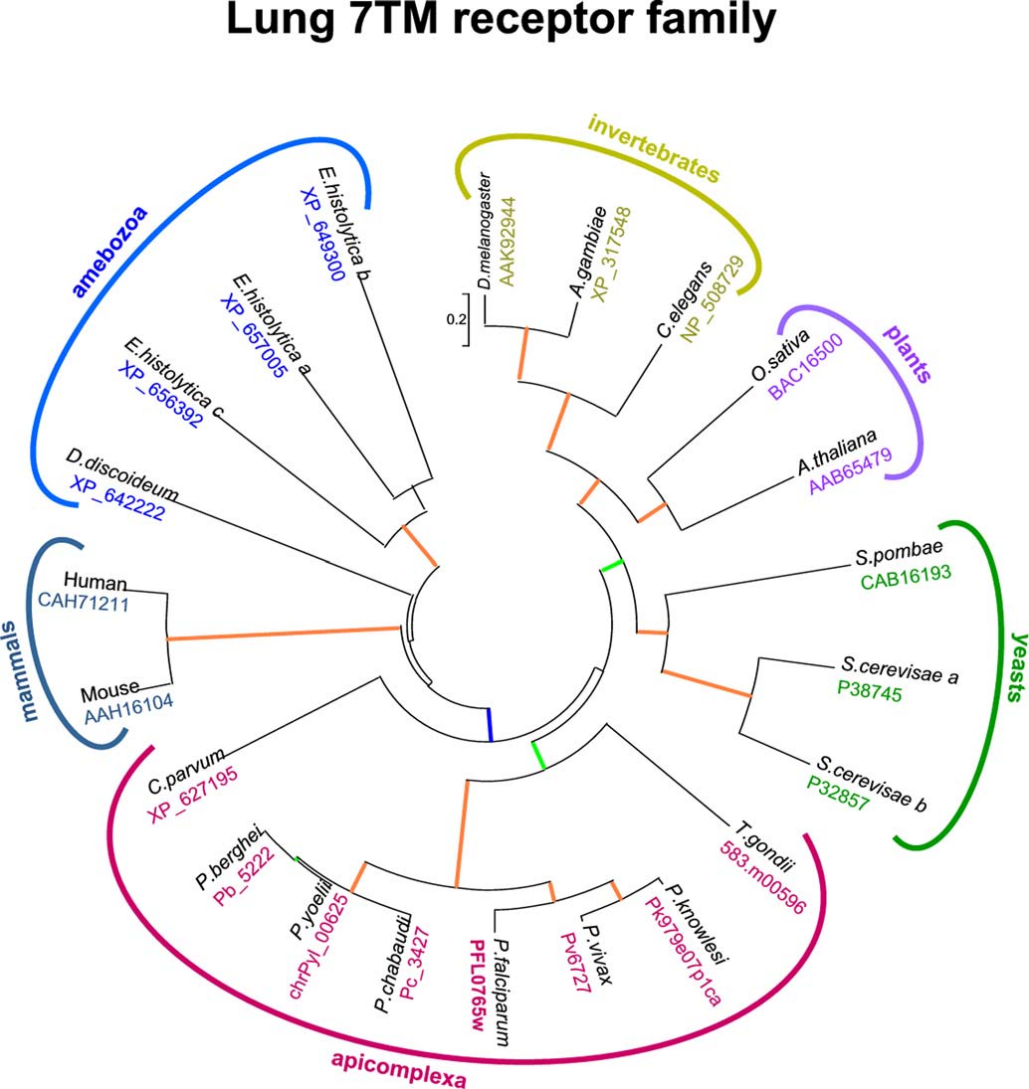
Phylogenetic tree of the evolutionary conserved “lung 7TM receptor” family. The tree was compiled using the aligned amino acid sequences of the 7TM conserved core region from several eukaryote organisms. Branches supporting bootstrap values >90% are in orange, >70% in green and >50% in blue (1000 replicates). Scale bar: 0.2 amino acid substitutions per site. Accession numbers shown correspond to the following databases: GenBank for non-*Plasmodium* members of lung 7TM receptor family; ToxoDB for *T. gondii*; PlasmoDB for Plasmodium species. Identification number for non-falciparum *Plasmodium* species are shown as contig numbers, and complete protein sequences are available in Data S2.

We next asked whether the serpentine receptor-like proteins are conserved in more distantly related organisms. We found that the 7-TM core region of PfSR10 (located in the C-terminal half of the protein, see Figure 2) is conserved among other protozoan parasites such as *Cryptosporidium parvum* and *Entamoeba histolytica*, as well as in unrelated organisms, such as *Saccharomyces cerevisae*, *Dictyostelium discoideum*, *Drosophila melanogaster*, *Caenorhabditis elegans* and *Arabidopsis thaliana* (Figure 4). Indeed, the region corresponding to the entire 7-TM core of PfSR10 has a Pfam score of 33.5 for the “lung seven-transmembrane receptor” conserved domain, found in all the related sequences of the other organisms mentioned above. This strongly supports the classification of PfSR10 in the heptahelical receptor family.

Initially, we did not identify significant homologues for the other PfSR-like proteins in non-*Plasmodium* organisms only by BLAST searches. However, by performing PSI-BLAST searches we found that PfSR12 has homologues with a novel class of orphan-receptors called intimal-thickness receptors or GPR180 [24]. Similarly, we found that PfSR1 shares similarity with cleft lip and palate transmembrane protein 1 (CLPTM 1), which function is still unknown [25]. On the other hand, by PSI-BLAST we found potential homologs for PfSR25 in three other Apicomplexa parasites (*Babesia bovis*, *Theileria annulata* and *Theileria parva*), but not in other organisms.

### Expression profile in the intraerythrocytic cycle

**Figure 5:**
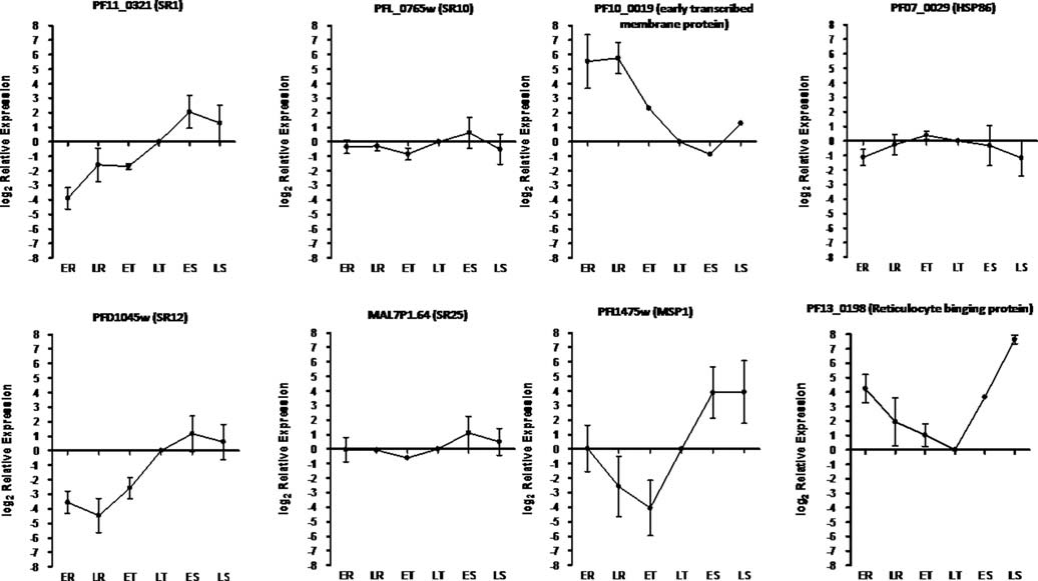
Expression of serpentine receptors in the *P. falciparum* intraerythrocytic cycle. Parasites cultures were synchronized with sorbitol and total RNA extracted from six time points during the 48 h intraerythrocytic cycle. Each RNA sample, converted to cDNA, was used as a template in a Real-time PCR reaction done in triplicates. The fluorescence values for each transcript were normalized by the ones of the 18S transcript and the expression is related to the one of the late trophozoite stage (LT). Error bars represent standard errors of two independent experiments. ER – early ring, LR – late ring, ET – early trophozoite, LT – late trophozoite, ES – early schizont, LS – late schizont.

To investigate whether the putative serpentine receptors are differentially expressed along the intraerythrocytic cycle, parasites were synchronized and RNA extracted from six time points during the intraerythrocytic development. Real-time PCR was applied to quantify the expression of the serpentine receptors in each intraerythrocytic stage. Two patterns of gene expression could be defined (Figure 5). *Pfsr1* and *pfsr12* are considerably more expressed in the second half of the intraerythrocytic cycle, from late trophozoite to schizont stages. On the other hand, *pfsr10* and *pfsr25* are constitutively expressed. As a control of parasites synchronization, the relative expression of genes well known to be either strongly regulated or constitutively expressed during the intraerythrocytic cycle was determined. As expected the expression profiles obtained from these genes, used here as markers for cycle progression, were very similar to data previously published [3], [4] and available at PlasmoDB.

## Discussion

### Searching for serpentine receptor-like proteins in malaria parasites

The *P. falciparum* genome is completely sequenced [18], the *P. yoelii* genome is extensively sequenced [18], [24], and currently a large number of genomic sequences from other *Plasmodium* species is also available. During the last years, a great deal of effort has been devoted to provide insight into stage-specific gene expression profile and function of the approximately 5300 genes of *P. falciparum*, 60% of which had no function assignments. Among the approaches used are DNA microarrays [3], [4], [25], serial analysis of gene expression [26] and mass spectrometry [6], [27], [28]. Moreover, systematic and extensive bioinformatics analysis of the parasite genome has identified the entire set of proteins involved in metabolic pathways, such as shikimate and pentose-phosphate pathways [29], [30], protein families, such as Rab GTPases [31], kinases [32], [33] and transporters [34].

Recently, a scan of genome sequences using a similarity search based on Hidden Markov Models constructed from the known serpentine receptors available in databases resulted in zero serpentine receptors in *P. falciparum* [35]. In contrast, a previous study identified and classified 46 putative serpentine receptors in the *P. falciparum* genome, based on predicted membrane topology patterns [17]. In view of this contradiction, we performed a thorough search for heptahelical receptors in the parasite genome. Since serpentine receptors are highly divergent at the amino acid sequence level [16], similarity-based searches are not indicated for finding new members of the heptahelic receptor family in such an evolutionarily distant organism as *Plasmodium*. So we decided to search for 7-TM proteins in *P. falciparum* genome.

We found four *P. falciparum* serpentine receptor-like proteins that show well-defined seven transmembrane domains (Figure 2). Indeed, the methodology applied by Inoue and colleagues [17] retrieved three of these heptahelical receptors; however, the use of redundant automatic gene predictions and of a different TM prediction program (HMMTOP) led these authors to a 10-fold overestimation in the number of putative serpentine receptors in the parasite. On the other hand, by using a series of stringent parameters as represented in Figure 1, we performed a careful analysis that enabled us to select four strong serpentine receptor-like candidates in *P. falciparum* genome, which account for just 5% of 7- and 8-TM proteins of the parasite.

### Diverse *Plasmodium* species possess serpentine receptor-like proteins

The four *P. falciparum* serpentine receptor-like proteins described here are currently annotated as hypothetical proteins. The 7-TM topology of the *Plasmodium* serpentine receptor-like proteins was confirmed by at least three other transmembrane prediction programs (Table 1). As to their probable sub cellular localization, PfSR10, 12 and 25 have cleavable N-terminal signal peptides, indicating that the N-terminus of these mature proteins must be on the extra-cellular side of the membrane, consistent with a serpentine receptor membrane topology. Importantly, *Plasmodium* has all the components necessary for the recognition and translocation of proteins containing signal peptides through the endoplasmic reticulum and also for the vesicle-trafficking apparatus responsible for their correct membrane targeting [5]. Although PfSR1 does not have a leading sequence, it is known that a large number of serpentine receptors lack a signal peptide but can still be efficiently translocated to the membrane [35].

By using real-time PCR we found that all four predicted serpentine receptors are transcribed and differentially regulated during the assexual intraerythrocytic cycle of *P. falciparum*. As a control of parasite synchronization and cell cycle progression, we determined the relative expression of the genes that encode for heat shock protein 86 (PF07_0029), early transcribed membrane protein (PF10_0019), merozoite surface protein 1 (PFI1475w) and reticulocyte protein 2 (PF13_0198). *Pfsr1* and *pfsr12* are up-regulated towards the late stages of the intraerythrocytic cycle, while *pfsr10* and *pfsr25* are constitutively expressed. Our results are in agreement with the microarray expression data available at PlasmoDB; only *pfsr12* has no array results in the public database. Moreover, according to the PlasmoDB microarray results, *pfsr1* and *pfsr25* are also expressed in gametocytes and sporozoites. Altogether, these data suggest that the putative serpentine receptors might have distinct functions required during specific stages of the life cycle.

We have also found homologues for the PfSRs in other *Plasmodium* species by using a homology-based gene prediction approach, and constructed a phylogenetic tree in order to verify their inter-relationships (Figure 3). Comparison of the amino acid sequences of the different PfSRs reveals that they are quite distinct from each other, so they do not seem to constitute a new family of serpentine receptors. However, they constituted individualized branches together with their homologues from other species. As expected, in all cases the sequences of rodent parasite receptors were grouped separately from the primate ones, and in the case of PfSR1 no homologues were found for rodent parasites. Phylogenetic studies performed with small subunit ribosomal RNA and cytochrome b genes revealed that the primate parasites *P. vivax* and *P. knowlesi* are closely related, while *P. falciparum* is even more distant from them than the rodent parasites *P. yoelii* and *P. berghei* [36], [37]. So, our findings may not reflect evolutionary relationships inside the *Plasmodium* genus, but raise the question of whether such proteins could be involved in parasite specific host infection.

Importantly, two of the herein identified *P. falciparum* serpentine receptors, PfSR10 and PfSR12, clearly showed homology with orphan G-protein coupled receptors that are found in diverse eukaryotic organisms, since yeast, plants, invertebrates and vertebrates. PfSR10 belongs to the family of 7-transmembrane lung receptors, while PfSR12 groups with a family of receptors related to the intimal thickening associated with vascular restenosis [24].

*Plasmodium* is a eukaryotic organism that displays different signaling pathways when compared to metazoan organisms and fungi, and its upstream signaling pathway components, *i.e.* membrane receptors, still remain unknown. Hence, the molecular identification of four putative serpentine receptor-like proteins in malaria parasites raises a new perspective on the signaling mechanisms operating in this protozoan. From now on, the challenge resides in the functional characterization of these putative receptors, in order to identify the PfSRs ligands and elucidate the signaling pathways in which they are involved.

## Materials and Methods

### Data base searches and sequence analysis

We performed the *P. falciparum* genome analysis for transmembrane proteins using three distinct approaches. In the first one, we retrieved the *P. falciparum* 3D7 genomic sequences available in PlasmoDB and extracted the intronless ORFs ≥500 bp long. A program was created to identify all of the intronless open reading frames (ORFs) present in the *P. falciparum* genome. Only ORFs larger than 300 nucleotides were identified. The program identifies an ATG and extracts the nucleotide sequence contained between this ATG and the next stop codon. The sequences were then translated and analyzed using TMHMM2.0 to select the ones showing seven predicted transmembrane helices [38], [39]. In parallel, the PlasmoDB annotated ORFs were analyzed using the ‘Gene Sequence Features – transmembrane domains’ tool available at this site, to search for sequences containing seven or eight transmembrane domains as predicted by TMHMM 2.0. The selected sequences were then submitted directly to the TMHMM 2.0 tool to recheck their topologies. Finally, the 46 *P. falciparum* putative serpentine receptor sequences identified by Inoue and colleagues [17] were analyzed using TMHMM 2.0, and the sequences with seven or eight transmembrane domains were selected and added to the sequences identified as explained above.

BLASTp and Pfam were used to scan the resulting sequences for the existence of conserved domains or similarity to non-serpentine receptor proteins [40], [41]. Low complexity sequences as well as sequences containing apicoplast transit peptides, as predicted by the PlasmoAP program (http://plasmodb.org/restricted/PlasmoAPcgi.shtml), or signal anchors, predicted by SignalP 3.0 [42], were eliminated. The selected *P. falciparum* serpentine receptor-like proteins were also analyzed using other TM prediction programs: TMAP, TOPPRED2, TMPRED and HMMTOP [43]–[46].

### Detection of serpentine receptor-like homologues

The PfSR amino acid sequences were used to search for homologues in other *Plasmodium* species, using tBLASTn against all *Plasmodium* species genomic sequences in the PlasmoDB site. The genomic sequences that matched each PfSR in the BLAST searches were retrieved along with their adjacent 5′ and 3′ flanking regions. These sequences were submitted to a HMM plus similar protein-based gene prediction using the FGENESH+ program [22] (http://www.softberry.com), setting the organism parameter as *P. falciparum*, in order to find the corresponding full-length ORF and determine their exon-intron gene structure. In further analyses, only the ORFs coding for the entire putative homologue proteins were used. For comparison, these sequences were submitted to transmembrane, signal anchor/peptide prediction as described above for *P. falciparum* proteins. Finally, PSI-BLAST searches were performed to investigate the existence of other homologous proteins in the non-redundant NCBI database.

### Sequence alignment and phylogenetic analysis

The extremely variable N-terminal and C-terminal regions were deleted from all *P. falciparum* serpentine receptor-like amino acid sequences. The sequences were then aligned using ClustalW version 1.8. The alignment was used as input to Mega2 [47] to construct a neighbor-joining tree from 1000 replicates of the interior branch test. Multiple alignments of PfSRs homologues amino acid sequences (shown in Data S3) were edited by Chroma [48].

### Real-time PCR

Total RNA was extracted from synchronized parasites in six time points during the intraerythrocytic cycle. Samples were treated with DNAse and then used in reactions for cDNA synthesis using Superscript II reverse transcriptase (Invitrogen) according to the manufacturer protocol. The SYBR green incorporation was measured during a PCR amplification carried on the 7300 Real Time PCR System (ABI) using the following cycling conditions: 50°C for 2 min, 95°C for 10 min, 40 cycles of 95°C for 15 sec; 55°C 30 sec; 60°C for 30 sec and ramp to 95°C at 0.2°C/sec. Dilutions of the cDNAs were used as standards in order to determine the amplification efficiencies of each gene. Only pairs of oligonucleotides with efficiencies between 90–110% were accepted and the efficiency was considered 100% for further relative expression quantifications. Water and no reverse transcriptase products were used as negative controls. The gene expression in each cDNA was normalized by the one of the 18S ribosomal RNA gene and expressed related to the expression in the late trophozoites stage (LT). The amplifications for each time point were done in triplicates with two independent parasite synchronizations and RNA extractions.

## Supporting Information

Data S1 Sequences of the primers used in RT-PCR to detect expression of PfSRs during intraerythrocytic cycle of P. falciparum and additional primers, used to amplify the full-length ORF of PfSRs
(0.05 MB DOC)

Data S2 Protein sequences of serpentine receptor-like homologues from Plasmodium species. They were obtained by using a homology-based gene prediction analyses from genomic sequences available in PlasmoDB, as detailed described in Materials and Methods
(0.04 MB DOC)

Data S3 Alignments of the four putative Plasmodium serpentine receptor-like proteins highlighting transmembrane domains predicted by TMHMM 2.0, signal peptides and potential cleavage sites predicted by SignalP 3.0, and sites of probable N-glycosylation and phosphorylation as predicted by NetNGlyc 1.0 and NetPhos 2.0 Servers, respectively
(0.20 MB DOC)

Data S4 Genomic sequences of four serpentine receptors of different species of malaria parasites. Sequences corresponding to introns are red-colored, and coding sequences are presented in black
(0.07 MB DOC)
