# Supplementary material for: Genome-Wide Detection of Serpentine Receptor-Like Proteins in Malaria Parasites
Source: PLoS One. 2008 Mar 26;3(3):e1889. doi: 10.1371/journal.pone.0001889 (PMC2268965; doi:10.1371/journal.pone.0001889)
Supplement: Data S1 — Sequences of the primers used in RT-PCR to detect expression of PfSRs during intraerythrocytic cycle of P. falciparum and additional primers, used to amplify the full-length ORF of PfSRs (0.05 MB DOC) [file pone.0001889.s001.doc]

**Supplementary data S1.** Multiple sequences alignment used for phylogenetic tree building. Only sequences corresponding to the 7-TM core regions were used. Multiple sequences alignment was done by ClustalW version 1.8.

#PbSR25(Pb_4740) ------------------------------------------------------------

#PySR25(PY01858) ------------------------------------------------------------

#PcSR25(Pc_5508) ------------------------------------------------------------

#PkSR25(Pk_347d07p1c) ------------------------------------------------------------

#PvSR25(Pv_6727) ------------------------------------------------------------

#PfSR25(MAL7P1.64) ------------------------------------------------------------

#PkSR10(Pk_979e07p1ca) ------------------------------------------------------------

#PvSR10(Pv_3938) ------------------------------------------------------------

#PfSR10(PFL0765w) ------------------------------------------------------------

#PySR10(chrPyl_00625) ------------------------------------------------------------

#PbSR10(Pb_5222) ------------------------------------------------------------

#PcSR10(Pc_3427) ------------------------------------------------------------

#PkSR1(Pk_279e09p1c) ------------------------------------------------------LIIHFC

#PvSR1(Pv_6737) NEMYSKDNHTQMGNVIEIEYSPISLPQFNLYNIIIFNVNYAKEKYKIAAYDLDSLVIHFC

#PfSR1(PF11_0321) ------------------------------------------------------ITTFLC

#PcSR12(Pc_6872) ------------------------------------------------------------

#PySR12(chrPyl_02193) ------------------------------------------------------------

#PfSR12(PFD1075w) ------------------------------------------------------------

#PkSR12(Pk_961e09q1c) ------------------------------------------------------------

#PbSR25(Pb_4740) -----WELLYVLTILNSLILIININYKENIDKLNNKKTEISDINDDLINVDINEFSNNER

#PySR25(PY01858) -----WELLYVLTILNSLLLMININYKENIDKLNNKKTEISDINDDLINVDINEFGNNER

#PcSR25(Pc_5508) -----WELLYVLTLLNIFILMLNVNYKENIDTLNNKKTEISDINDDLINVDINEFGNNER

#PkSR25(Pk_347d07p1c) ---VKWELLGVLTILNSLILLLNVNYKENINHLNNKKSETSDINDDLINVDMNEFSNNEK

#PvSR25(Pv_6727) ------ELLGVLTILNSLILLLNVNYKENINHLNNKKSETSDINDDLINVDMNEFSNNEK

#PfSR25(MAL7P1.64) ------ELLALLTVVNMLLLLLNVNYKENINHLNNKKSETSDINDDLINVDMHEFSNNEK

#PkSR10(Pk_979e07p1ca) ---------LNLYVVCMLIY----------------------------------------

#PvSR10(Pv_3938) ---------LNLYVLCMIIY----------------------------------------

#PfSR10(PFL0765w) ---------LNLYVLSMIIY----------------------------------------

#PySR10(chrPyl_00625) ---------LNLYVSLMLIY----------------------------------------

#PbSR10(Pb_5222) ---------LNLYVSLMIIY----------------------------------------

#PcSR10(Pc_3427) ---------LNLYVSLMIIY----------------------------------------

#PkSR1(Pk_279e09p1c) GNIYLCLIIFILCFILLLMDLMAL------------------------------------

#PvSR1(Pv_6737) GNISLCLIICLLCLILLLIDLVAL------------------------------------

#PfSR1(PF11_0321) CHITCSMIIYILCIIYIIMEITYL------------------------------------

#PcSR12(Pc_6872) -----------LIEIYMLLFVIST------------------------------------

#PySR12(chrPyl_02193) -----------LIEISMLLFVILS------------------------------------

#PfSR12(PFD1075w) -----------LFEIHMLLIVLLF------------------------------------

#PkSR12(Pk_961e09q1c) -----------LIEIHFLLVVILV------------------------------------

#PbSR25(Pb_4740) DDSDDELEKEKRYNKLKIKNLYSISNSRICYYSMWILCYYFIYFLCFLSFLYGIRLFNNN

#PySR25(PY01858) DDSGDELEKEKRYNKLKIKNLYSISNSRICYYSMWTLCYYCIYFLCFLSFLYGIRLFNNN

#PcSR25(Pc_5508) DDSGDELEKEKRYNKLKIKTLYSITNSRICYYSMWILCYYSIYFLCFLSFLHGIRLFNNN

#PkSR25(Pk_347d07p1c) DESSDESEKEKKYNKIKIRYLYSISNSRVCYYSMWILCYYLIYFLCFLSFLYGIRLFQNN

#PvSR25(Pv_6727) DESSDESEREKKYNKIKIRYLYSISNSRVCYYSMWILCYYLIYFLCFLSFLYGIRLFNNN

#PfSR25(MAL7P1.64) DESSDENEKEKKYNKLKIRYLYNVSNSIICYYSLWILCYYLIYFLCFLSFLYGIRKFNNN

#PkSR10(Pk_979e07p1ca) -------------AIYLFTWIYLLIRNKQFVIKIQIWILVCTFLYLMENVFLFLYFLVYN

#PvSR10(Pv_3938) -------------AIYLFAWIYLLMRNKQFVIKIQIWILVCIFLYLLENFFLFLYFLVYN

#PfSR10(PFL0765w) -------------SIYLFIWSYLLIRNKNYVIKIQIWILVCVFLYLIENICLFLYFLSYN

#PySR10(chrPyl_00625) -------------FIYSIIWSYSLIKNKANVIKIQVWISVCIFLYLLENLFLYLYFMTYN

#PbSR10(Pb_5222) -------------FIYSMIWSYSLIKNKTNVIKIQVWISVCIFLYLLENMFLYLYFMTYN

#PcSR10(Pc_3427) -------------LLYSIIWSYSLFKNKTNVIKIQVWISVCMLLYLIENIFLYLYFMTYN

#PkSR1(Pk_279e09p1c) -----------LFDWSRWNRVNDLYSFPS-YTVHFKLIFTLFIFLYLKNKNSCNILMVFC

#PvSR1(Pv_6737) -----------VFDLTSWNRLNGLYSFSS-DALHFKFLFSMFIFLYLKNKSNCKILMVFC

#PfSR1(PF11_0321) -----------LFDIKMWKRWNNLYTFTYNNDIVMNITLLFFILLYLRNIDYGRVIMIYY

#PcSR12(Pc_6872) -----------VLSLVYVRKRSMLNNANG---ALKESVHFGVLFFYFSNIFYLIHIYSYA

#PySR12(chrPyl_02193) -----------VLSLIYFRKRNMLSKENG---TLKESVHSGALFFYFSNIFYLIHIYAYA

#PfSR12(PFD1075w) -----------VLSLVYYRKRKNLNNTNN---VLKEAIHCSYLFFLLSNILYFIHLISYA

#PkSR12(Pk_961e09q1c) -----------VLSLAYKSRQDSLRGAHS---AMKEGIHMSVMFFVLSNLCYLIHIFFYA

#PbSR25(Pb_4740) LINIYTIRTCKIEKLENYIISENTFTSLYWVFIN------------------FNVFMSKY

#PySR25(PY01858) LINIYTIRTCKIEKLENYIISENTFTSLYWVFIN------------------FNVFMSKY

#PcSR25(Pc_5508) LINIYTIRTCKLEKLNNYIISENTFTSLYWVFIN------------------FSVFMSKY

#PkSR25(Pk_347d07p1c) LINIYTIRTCRIDNLANYILSENTFISLYWAIIN------------------FNVFMSKY

#PvSR25(Pv_6727) LINIYAIRTCKIDNLANYILSENTFISLYWAIIN------------------FNVFMSKY

#PfSR25(MAL7P1.64) VINIYTLRTCKIDKLTNYILSENTFISLYWAIIN------------------FNVFMSKY

#PkSR10(Pk_979e07p1ca) LRARVNSNLLFLSVCSSILKNVCSYLLILLGSLG------------------WGLVIPTL

#PvSR10(Pv_3938) IRARVNSNLLFLSVCTSILKNVCSYLLILLGSLG------------------WGLVIPTL

#PfSR10(PFL0765w) LYAKVNNELLFISVCSSILKNVCSYLLILLGSLG------------------WGIVIPTL

#PySR10(chrPyl_00625) VQAKINNNYLFMAVFFSVLKNVCSYLLILLGSLG------------------WGLVIPTL

#PbSR10(Pb_5222) VQAKINNNYLFMAVFFSVLKNVCSYLLILLGSLG------------------WGLVIPTL

#PcSR10(Pc_3427) VQAKINNNYLFMAVFFSVLKNVCSYLLILLGSLG------------------WGLVIPTL

#PkSR1(Pk_279e09p1c) VAKMAVCLWKLLDHYDIEFMEIHPYVRITRNI--------EEISNIGE-RGNEN------

#PvSR1(Pv_6737) VAKMAVCLWKLLDRYDIEFMEVHPYVCITSSTGGGAGSAGSARSGVGGGSGNDNPGNGGG

#PfSR1(PF11_0321) IMKMMVLIFKIIYNYDICILNDYPYICMNKKS----------------------------

#PcSR12(Pc_6872) FNGTGFSILKVLSQIYESIFDCITLTIIFYIVNT--------------------------

#PySR12(chrPyl_02193) LNGAGFSVLKVLSQIYESIFDCIILTILFYLINS--------------------------

#PfSR12(PFD1075w) FNGSGFSILKVLSQIYEAIFDCFILVIIYYIFN---------------------------

#PkSR12(Pk_961e09q1c) FDGTGLTSLKVLSQMGESIYDCFVMTIIFYIMCC--------------------------

#PbSR25(Pb_4740) TDSFYAINYVNFNIEFSTKKKRTLFFLNYAYQLLLITYSIYKNVLLYKRG----------

#PySR25(PY01858) TDSFYAINYVNFNIEFSTKRKRTLFFLNYAYQLLLITYSIYKNVLLYKKG----------

#PcSR25(Pc_5508) TDSFYAINYVNFNIEFSTKRKRTLFFLNYAFQLLLITYTIYKNVLLYQNG----------

#PkSR25(Pk_347d07p1c) TDSFYISNYFKLNIQFSTGKKKLLFFLNYAYQILLVSYTIYKNVDLYNKG----------

#PvSR25(Pv_6727) TDSFYISNYIKLNLEFTTGKKKLFFFVNYAYQILLLSYTIYKNVSLYNKG----------

#PfSR25(MAL7P1.64) TDSFYVVNYFKLNFEFSNRKKKTLFILNYMYQLLLLSYTIYKNITLYTKG----------

#PkSR10(Pk_979e07p1ca) DKKTFIK--IKVLFFFFIIFDFIKQFLDMHLTDAEVNAVYFLFCIIPVTI----------

#PvSR10(Pv_3938) DKKTFIK--IKVLFFFFIIFDFIKQFLDMHLTDAEVNAVYFLFCIIPVTI----------

#PfSR10(PFL0765w) DRKTFIK--IKILFFFFIIFDFIKQFVDMHLTDTQINTGYFFFCIIPVTI----------

#PySR10(chrPyl_00625) DRKTFIK--IKVLFIFFIIFDFIKQFLDAHLADEHVNTVYFLCCILPMSI----------

#PbSR10(Pb_5222) DRKTFIK--IKVLFIFFIIFDFIKQFLDAHLAEEHVNAVYFLCCILPMSI----------

#PcSR10(Pc_3427) DKKTFIK--IKVLFIFFIIFDFIKQLLDAHLAEEHVNTVYFLCCILPMSI----------

#PkSR1(Pk_279e09p1c) --QSIDKGAIIQKNQFRTELENIETYIKMKMPNVMFCTVVSMCAYNFIYT----------

#PvSR1(Pv_6737) SGSGSGGGAGTHKRQSRSEVEDLERYIKMKMPNVMICTVVSTCAYNFMYT----------

#PfSR1(PF11_0321) ---------LKEMNKEMIMDEEFEKKIKKKVNIFMIFSIILIFIYNYFYT----------

#PcSR12(Pc_6872) ------INNKKRRKEDTIKTGFIYSILKFFYILFEMQNHQSLNVYSSLHS----------

#PySR12(chrPyl_02193) ------IHNKKKRKEDTIKTGFIYSMLKFFYILFEIQNHQTLNAYSSLHS----------

#PfSR12(PFD1075w) -------NDMQKKKEETIRVAFTYSILKFIYILFEIQNNQELDLYSTLHS----------

#PkSR12(Pk_961e09q1c) ------TMDREKRRKDTFRTALNYGVLKFLYLLVEMQNQEDLNLYASLHSGWGGLANYGT

#PbSR25(Pb_4740) --------------LYNLNQIVCSIIFLCLILYTIFEITYVLEINKPSYYSMPKLSYNYV

#PySR25(PY01858) --------------LYNLNQIVCSLIFLCLILYTIFEITYVLEINKPSYYSMPKLSYNYI

#PcSR25(Pc_5508) --------------VYNLNQIVCSLIFLCLILYTIFEIAYVLEINKPSYYSMPKLPYNYV

#PkSR25(Pk_347d07p1c) --------------LYNLNQIVCALIFLCLILYTILEITYVLEINKPCYYGVTKLSFNYI

#PvSR25(Pv_6727) --------------LYNLNQIVCALIFLCLILYTILEITYVLEINKPCYYGVTKLSFNYI

#PfSR25(MAL7P1.64) --------------EYNLNQIICALIFLCLILYTILEITYVLEINRPCYNVQTKLPFHYV

#PkSR10(Pk_979e07p1ca) --------------IYSIIYLWVFTSASKIIIQLNEDKQYEKLNMFKKFFNV--LIFSLI

#PvSR10(Pv_3938) --------------IYSIIYLWVFTSASKIIIQLNEDKQYEKLNMFKKFFNV--LIFSLI

#PfSR10(PFL0765w) --------------IYSIIYIWVFTSASQIIIQLNEDKQYEKLNMFKNLFNV--LIFTLL

#PySR10(chrPyl_00625) --------------IYAIIYVWIFISSSKIIIQLNEDKQYEKLNMFKNFFNV--LILALI

#PbSR10(Pb_5222) --------------IYAIIYIWIFISSSKIIIQLNEDKQYEKLNMFKNFFNV--LILALI

#PcSR10(Pc_3427) --------------IYSIIYMWVFISSSKIIIQLNEDKQYEKLNMFKNFFNV--LILALI

#PkSR1(Pk_279e09p1c) ----------------QYESIYAFIIHSIAICSYIFNFVFMCPQIVRNYYTRTVERVPLF

#PvSR1(Pv_6737) ----------------QYDSVYAFIIHSVAVCSYIFNFLFMCPQIVRNYHTKTVERVPLF

#PfSR1(PF11_0321) ----------------KYDSYYSYVIHTLGFSSYLYKFILMLPQIITNIYTRTVQRMSFP

#PcSR12(Pc_6872) ------------------VVALPFVSHRVIISVLIYNNCKKLLKEKTSASDKTRLLLDAS

#PySR12(chrPyl_02193) ------------------VVAFPFVSHRVFYMKLFY------------------LVLFGF

#PfSR12(PFD1075w) ------------------IVALPFVVYRIIVAVLNYDNSKKLLKEKTQVDEKFYVLFDTF

#PkSR12(Pk_961e09q1c) VTSRVEEMPTPSYSLPLSLVALPFVLYRVIIAATIYRNYKRLLMEKTSREETFFISLHMF

#PbSR25(Pb_4740) WS-------IIYLFVIFISSVIFYFSVYSYSIKDTFVNFQTMLWLFFISLTYI-------

#PySR25(PY01858) WS-------IIYLFVIFISSVIFYFSVYAYSIKDTFVNFQIMLWLFFISLTYI-------

#PcSR25(Pc_5508) WS-------IIYLFVIFVSSVIFYFSVYAYSIKDTFVNFQIMLWLFFLSLTYI-------

#PkSR25(Pk_347d07p1c) WA-------IIYLFVIFISSVIFYFSVFPYSIKDQYVNFQIMLWFFFISLTYI-------

#PvSR25(Pv_6727) WA-------IIYLFVIFISSVIFYFSVFSYSIKDQFVNFQIMLWFFFISLTYI-------

#PfSR25(MAL7P1.64) WA-------IIYLFIIFTSSVIFYFSVFSYSIKDQFVNFQITLWLFFISLTYI-------

#PkSR10(Pk_979e07p1ca) FS-------VISFVIDIVVMLFVDNTIWSLKCYISEGIISCLFLIIITAMFMLFR-----

#PvSR10(Pv_3938) FS-------VIAFVIDIVVMLFVDNTIWSLKCYLSEGIISCLFLIIITAMFMLFR-----

#PfSR10(PFL0765w) FS-------VIAFIIDIVVMLYVDNSIWNLKNYLSEGIISCLFLIILTAMFILF------

#PySR10(chrPyl_00625) FS-------IISLIIDLFVMLFPSDQLWNLKCYISEGVNSFLFLTVLTAMCMLF------

#PbSR10(Pb_5222) FS-------IISLIIDLFVMLFPSDQLWNLKCYISEGVNSFLFLTVLSAMCMLFKPSERL

#PcSR10(Pc_3427) FS-------IISLIIDLFVMMFPNEQLWNLKCYISEGVNSCLFLTVLTAMCVLF------

#PkSR1(Pk_279e09p1c) F------LFFLFLYALMDDLFVLVLRIPLVHKWNALGDDIVFFIFFVQYCVY--------

#PvSR1(Pv_6737) F------FFFLFLYAIMDDLFA--------------------------------------

#PfSR1(PF11_0321) F------FLFLLVNVLINDLFIIFLRMPKVHKYYLFADDFILFLFIIQYCIY--------

#PcSR12(Pc_6872) M------YEPKKKKAMSQWGVYRMDSLYSVYLFFFVECFNALYPFVHSF-----------

#PySR12(chrPyl_02193) I------FEG------------------LVY-----------------------------

#PfSR12(PFD1075w) F------YNLWILSIPVQYFLMKSFSLHFTHLFVHFFNLYILIYLVYNLS----------

#PkSR12(Pk_961e09q1c) LLVNEEGYNLWILSIPAYYLLMSRASIFGAYHFGEEVRSHGIQASLLGYG----------

#PbSR25(Pb_4740) ----------

#PySR25(PY01858) ----------

#PcSR25(Pc_5508) ----------

#PkSR25(Pk_347d07p1c) ----------

#PvSR25(Pv_6727) ----------

#PfSR25(MAL7P1.64) ----------

#PkSR10(Pk_979e07p1ca) ----------

#PvSR10(Pv_3938) ----------

#PfSR10(PFL0765w) ----------

#PySR10(chrPyl_00625) ----------

#PbSR10(Pb_5222) KRISHFTEIG

#PcSR10(Pc_3427) ----------

#PkSR1(Pk_279e09p1c) ----------

#PvSR1(Pv_6737) ----------

#PfSR1(PF11_0321) ----------

#PcSR12(Pc_6872) ----------

#PySR12(chrPyl_02193) ----------

#PfSR12(PFD1075w) ----------

#PkSR12(Pk_961e09q1c) ----------
