# Supplementary material for: Genome-Wide Detection of Serpentine Receptor-Like Proteins in Malaria Parasites
Source: PLoS One. 2008 Mar 26;3(3):e1889. doi: 10.1371/journal.pone.0001889 (PMC2268965; doi:10.1371/journal.pone.0001889)
Supplement: Data S2 — Protein sequences of serpentine receptor-like homologues from Plasmodium species. They were obtained by using a homology-based gene prediction analyses from genomic sequences available in PlasmoDB, as detailed described in Materials and Methods (0.04 MB DOC) [file pone.0001889.s002.doc]

**Supplementary data S2:** Protein sequences of serpentine receptor-like homologues from *Plasmodium* species. They were obtained by using a homology-based gene prediction analyses from genomic sequences available in PlasmoDB, as detailed described in Materials and Methods.

>PfSR1

MIKIIIGVIGYYILYSSYHIYENIKSPIFNDGNVIKNNEGDKNEQLVIPKEGKKRNSINEEEEEYINKPFKNVLKKDDIIDYHLYFSCEEDIDINKHMQDKYLEKDERFISVYKILNGKYSWNNNVDLLDEKRKKNSFFFLFEKVHPSFDISIPKELIDQRKDIYLHILTYVNKELYRYGSRTVVITKRKEKTTTRGKKKFLWKSLIDEEEEEEEEEEEEEEEEEEEEEEEEEEEEEEEEEEEEEEEKKYQHGNYEKGGSYKVHKNISNNKPNNKNPNDKRDIKHNNNNNNNKMKKDNFIKSNQNSLNIHKKKKKRKHKDFLFYIPKKIRFGPVIEYNDFHISKLGFFSNMHVDKDTNTYLLPIYINNDLTPDDEYRMIKMKNSDDMMKKKLKKKRSSEKNDPYYKENVRNDNNNNNNNNNIKNNYLEIYDLSKRTYEMRNERNKKIYEKNSSINNNVHKLEHELMDYIINIEYVPINYNYYNLLNMLKFNVSYVKKKYNFISFDMDSITTFLCCHITCSMIIYILCIIYIIMEITYLLFDIKMWKRWNNLYTFTYNNDIVMNITLLFFILLYLRNIDYGRVIMIYYIMKMMVLIFKIIYNYDICILNDYPYICMNKKSLKEMNKEMIMDEEFEKKIKKKVNIFMIFSIILIFIYNYFYTKYDSYYSYVIHTLGFSSYLYKFILMLPQIITNIYTRTVQRMSFPFFLFLLVNVLINDLFIIFLRMPKVHKYYLFADDFILFLFIIQYCIYKKENKIFGAREKLVLLKNAKKNK

>PkSR1

MIKFAVGIICYYIVYCAYHVYQNVRTPIYEYAKDVKKNEKDQEGLHVGEKYIHRAFTNFLKQKDKVDYHLYMSCEENIDLNRYVSEKDKFLEKNKNFINVHNFNNISYDWNYSDPVEGETKWWNFLSPRKYRSVEVTIPSSLIKKNKEIYLHILTYVNGELYRHGFVTSILTREKVGAKKSTAKEKYLWERLLEEEEEMDDYNDDGNDDMEESEDDNDNDDEDEENEQKTQTDDSSNDRQRKSVSKQTQEGVKKKNNKMKAKKRPVKKKKKKKKKNFYIPKKVKFGPVIEHNDINVNKIGFFSNIFLDTENSVYLLPTYYNDHLTPEDEYELLQIVWEGEKEDVIARKRKKNNTQHDHVTKSDDNYTHRGNIIEIELSPISLPQFNLYNIILFNINYAKEKYKFVTYDLDNLIIHFCGNIYLCLIIFILCFILLLMDLMALLFDWSRWNRVNDLYSFPSYTVHFKLIFTLFIFLYLKNKNSCNILMVFCVAKMAVCLWKLLDHYDIEFMEIHPYVRITRNIEEISNIGERGNENQSIDKGAIIQKNQFRTELENIETYIKMKMPNVMFCTVVSMCAYNFIYTQYESIYAFIIHSIAICSYIFNFVFMCPQIVRNYYTRTVERVPLFFLFFLFLYALMDDLFVLVLRIPLVHKWNALGDDIVFFIFFVQYCVYKKGKSRVGSGEVAAAPQGAKPQRESKKKK

>PvSR1

MIKFAVGIICYYIVYGSYHVYQNVRTPVYDHSRDGKESGKEQEGPHVGEKYIHRPFRNFFKQKDKVDYHLYMSCEEDIDLNRYVNEKEKYLKKNKNFINVYNFNNVSYDWSYSHPAGGGGNKWWNVLSPRKYPSVKLTIPRKLIKKNKEIYLHIITYVNGELYRHGFVTSALTRGKRGGKKWTRKEKYLWERFLDEEEDDEEEEEEEEEEEEEDDEVDEAYNGQYGGHYTGEYNEQHAEQHADDAEGHNEAEEGDDDDDDEEEEEKEAEGENDEETQTGDASNEGQKKSSGVATHEGVKKKNKKMKAKKRHAKKTKQKIFYIPKKVKFGPVIEHNDINVNKIGFFSNIFLDKENSVYLLPTYYNDHLTAEDEYELLQIGGEGEKEDVGKKKKKKNAQEDNEMYSKDNHTQMGNVIEIEYSPISLPQFNLYNIIIFNVNYAKEKYKIAAYDLDSLVIHFCGNISLCLIICLLCLILLLIDLVALVFDLTSWNRLNGLYSFSSDALHFKFLFSMFIFLYLKNKSNCKILMVFCVAKMAVCLWKLLDRYDIEFMEVHPYVCITSSTGGGAGSAGSARSGVGGGSGNDNPGNGGGSGSGSGGGAGTHKRQSRSEVEDLERYIKMKMPNVMICTVVSTCAYNFMYTQYDSVYAFIIHSVAVCSYIFNFLFMCPQIVRNYHTKTVERVPLFFFFFLFLYAIMDDLFALVLRMPLVHKWNALGDDLVFFIFLLQYCVYKKGDSRVAPGEAPAAPPGTKAQRECKKRK

>PfSR10

MVIWKGNVKNKILFLIFVAYFFVFVKISNGQLIKLDGQKINTNYILYVLKGLYIFGENESPYVLLGKKKDMDFKAAHAIFENVGISTTDNKNTKYFSFEMGDTTSGNNENNNNNNNDGHNNNNNDSHNNNNNDGHNNNYDHNNDSTLENTNLPQNSYNNNGNNGNNSSEKHKDEDEDKDKFKINLYKDNPYLRKKKEYRYSEDVDSFVTSELFLELIIMKEKDFNKHYLPKDHDICCYMQEEGIDGYEKYTCPGKGYLKRYVDEEHMYSLKLPVYFINDRIKDDTNNNNNNNNNNNNNNSSSSSSSSYYNNMYNLNNGNEINHENLINHLKNKFVYNIKDTDVYALFLSNCLDSKKYELHLHGNIHILNDYGYLPGDKISKLNLYVLSMIIYSIYLFIWSYLLIRNKNYVIKIQIWILVCVFLYLIENICLFLYFLSYNLYAKVNNELLFISVCSSILKNVCSYLLILLGSLGWGIVIPTLDRKTFIKIKILFFFFIIFDFIKQFVDMHLTDTQINTGYFFFCIIPVTIIYSIIYIWVFTSASQIIIQLNEDKQYEKLNMFKNLFNVLIFTLLFSVIAFIIDIVVMLYVDNSIWNLKNYLSEGIISCLFLIILTAMFILFKPSDRLKRISHFTEIGDMDEMEDFSNFKNSIEDIS

>PkSR10

MIWKSTQKVKCKTEFLIVVFLVLFASITNSQLIKLDGQKISTNYILYVLKGLYIFGKNDTPYVLLGEKKDMATKGPHAIFENIGISTTDNKNTKYFSFDIEESRTNGQENNESDEENKSDEHPTSEMNHSSDGDDDDDDEKDKKDKFKINLYKDNPYLRKKKEYMHKHEDDEVLDAGNLFLEVIIMKESDFNKFYLPKDTNVCCHMEENGMDGNDSYTCPGRGYLKRYVEESSMYSLKLPVYFLNDRISNDDEISPLENEVNHENFLKKIQNKHIYNIDDTDVYALFLSNCSDSKKYELELHGNIHILNKYGYLPGDKIPKLNLYVVCMLIYAIYLFTWIYLLIRNKQFVIKIQIWILVCTFLYLMENVFLFLYFLVYNLRARVNSNLLFLSVCSSILKNVCSYLLILLGSLGWGLVIPTLDKKTFIKIKVLFFFFIIFDFIKQFLDMHLTDAEVNAVYFLFCIIPVTIIYSIIYLWVFTSASKIIIQLNEDKQYEKLNMFKKFFNVLIFSLIFSVISFVIDIVVMLFVDNTIWSLKCYISEGIISCLFLIIITAMFMLFRPSDRLKRISHFTEIGDMDEMEDFSQFKGSIEDIS

>PvSR10

MMIWKSTQKVKCKSEFLIVVFLLLFASITSSQLIKLDGQKINTNYILYVLKGLYIFGKNDSPYVLLGEKKDMATKGPHAIFENIGISTTDNKNTKYFSFDMEENKSDEEHKADEHSTSESNNGSDDEDEDSDEKDKKDKFKINLYKDNPYLRKKKEYMHKHEEDEVLNTGNLFLEVVIMKESDFNKFYLPKDSNVCCHTEERGMDGNDAYTCPGRGYLKRYVKESSMYALKLPVYFLNDRISNNDGMSPLENEVNHEEFLKKIQSRHVYNIDETDVYALFLSNCSDSKKYELELHGNIHILNKYGYLPGDKIPKLNLYVLCMIIYAIYLFAWIYLLMRNKQFVIKIQIWILVCIFLYLLENFFLFLYFLVYNIRARVNSNLLFLSVCTSILKNVCSYLLILLGSLGWGLVIPTLDKKTFIKIKVLFFFFIIFDFIKQFLDMHLTDAEVNAVYFLFCIIPVTIIYSIIYLWVFTSASKIIIQLNEDKQYEKLNMFKKFFNVLIFSLIFSVIAFVIDIVVMLFVDNTIWSLKCYLSEGIISCLFLIIITAMFMLFRPSDRLKRISHFTEIGDMDEMEDFSHFKASMEDIS

>PbSR10

MVIWKANPKNKNLLYLLFLYIFFISFTNCQLIKLDGQKINTNYILYVLKGLYIYGKNDVPYILLGEKKDMNSAGPHAIFENVGISTTEIKNTKYFTFGRKNNSDHNDNANEKNDEDGEMEKTNNNNSTSYEEEDDESEEKRKKKGFKLNLYKDNPYVQRKTEHSDLEDWDSNDNISDLFLEIIIMKESDFNKLYLPKDSNMCCYTEMTGIDNNDKYTCPGKGYLKRYLGESEFHSLKVPIYFINDRIEDDNTSSGNEVNYNKFLELIKNEHIYNIDKTDIYTVFLSNCGDSKVYELDLHGNIHILNKYGYLPGDKIPKLNLYVSLMIIYFIYSMIWSYSLIKNKTNVIKIQVWISVCIFLYLLENMFLYLYFMTYNVQAKINNNYLFMAVFFSVLKNVCSYLLILLGSLGWGLVIPTLDRKTFIKIKVLFIFFIIFDFIKQFLDAHLAEEHVNAVYFLCCILPMSIIYAIIYIWIFISSSKIIIQLNEDKQYEKLNMFKNFFNVLILALIFSIISLIIDLFVMLFPSDQLWNLKCYISEGVNSFLFLTVLSAMCMLFKPSERLKRISHFTEIGDMDEMDDFSHFKNSIEDIS

>PcSR10

MGIWKANPKNKNLLYLLFLYIFFISFTNCQLIKLDGQKINTNYILYVLKGLYIYGKNDAPYILLGEKKDMNNAGPHAIFENVGISTNEIKNTKYFSFGMKHDSHHDDNINEKHNEDGEAEKAKNHNHNSYDDEDDESDENNKKKGFKLNLYKDNPYVQRKTEHSETKSWNSNDNTSDLFLEIIIMKETDFNKLYLPKDTKRCCYTKMTGFDNSDKYTCPGKGYLKRYLDESEMHSLKVPIYFINDRIEDNDTSSGNEVNHNKFLELIKNEHVFNIDKTDIYTVFISNCGDSKIYELELHGNIHILNKYGYLPGDKITKLNLYVSLMIIYLLYSIIWSYSLFKNKTNVIKIQVWISVCMLLYLIENIFLYLYFMTYNVQAKINNNYLFMAVFFSVLKNVCSYLLILLGSLGWGLVIPTLDKKTFIKIKVLFIFFIIFDFIKQLLDAHLAEEHVNTVYFLCCILPMSIIYSIIYMWVFISSSKIIIQLNEDKQYEKLNMFKNFFNVLILALIFSIISLIIDLFVMMFPNEQLWNLKCYISEGVNSCLFLTVLTAMCVLFKPSERLKRISHFTEIGDMDEMDDFSHFKNSIEDV

>PySR10

MVIWKANPKNKNLLYLLFLYIFFISFTNCQLIKLDGQKINTNYILYVLKGLYIYGKDDAPYILLGEKKDMNNAIPHAIFENVGISTNEIKNTKYFTFGRKNNSDHDDNVNEKNDEDGEMEKVNNNNSTSYEEDDESEEKKKKKGFKLNLYKDNPYVQRKTEHSDLEDLDSNDNTSDLFLEIIIMKESDFNKLYLPKDSNMCCYTEMTGVDNNDKYTCPGKGYLKRYLDESEMHSLKVPIYFINDRIEDDNTSSGNEVNHNKFLELIKNEHIYNIDKTDIYTIFLSNCGDSKIYELDLHGNIHILNKYGYLPGDKITKLNLYVSLMLIYFIYSIIWSYSLIKNKANVIKIQVWISVCIFLYLLENLFLYLYFMTYNVQAKINNNYLFMAVFFSVLKNVCSYLLILLGSLGWGLVIPTLDRKTFIKIKVLFIFFIIFDFIKQFLDAHLADEHVNTVYFLCCILPMSIIYAIIYVWIFISSSKIIIQLNEDKQYEKLNMFKNFFNVLILALIFSIISLIIDLFVMLFPSDQLWNLKCYISEGVNSFLFLTVLTAMCMLFKPSERLKRISHFTEIGDMDEMDDFSHFKNSIEDIS

>PfSR12

MIRRKWSKIKLAIYFIAFYYLTKIDEKCLLIKEGELNLSITNVHDNNYMISEKYNKYYILSLLYKFGGILKEIYDKKIFVKNKSCNNFMVSSKVIYGLYNDMNYSKYSNFCFSKNANNGVVILSNLYVPNTKFLILDKSDDEIYNYGKNKNGKTCEDLEKIALFVHPLNDIPPELMNKTYFVYQKDIEKSLTDKKLNFILLNCGNKIKNAFKIEFKNNMNFLKNHFSCEEQGLFEIHMLLIVLLFVLSLVYYRKRKNLNNTNNVLKEAIHCSYLFFLLSNILYFIHLISYAFNGSGFSILKVLSQIYEAIFDCFILVIIYYIFNNDMQKKKEETIRVAFTYSILKFIYILFEIQNNQELDLYSTLHSIVALPFVVYRIIVAVLNYDNSKKLLKEKTQVDEKFYVLFDTFFYNLWILSIPVQYFLMKSFSLHFTHLFVHFFNLYILIYLVYNLSEEKFEVLESKHPYLDLN

>PkSR12

MAMPKGGFLTVLFFLLCIAHWDGGFPPPDRDCRQSCPKLCPTDGESYTWGIWPRSSVPMMNVKGRSIASGFYATGKVIYGLKEERNFSVYGDFCYSRSRHTMGRNGVLIVTSSYIPNTKLLILKKVEEDINNYMNGKNGNRCADLERKALFVHPFDNTPEGNLPNSYSLYEEDIGDNLKDTPLNFVLLACGRHVKNAYKIEFRNNAHFLRNHFSCEDQGLIEIHFLLVVILVVLSLAYKSRQDSLRGAHSAMKEGIHMSVMFFVLSNLCYLIHIFFYAFDGTGLTSLKVLSQMGESIYDCFVMTIIFYIMCCTMDREKRRKDTFRTALNYGVLKFLYLLVEMQNQEDLNLYASLHSVVALPFVLYRVIIAATIYRNYKRLLMEKTSREETFFISLHMFLYNLWILSIPAYYLLMSRASIHFTHLYVHFLNLLILIYLVHIISEKKYEVMESRHPYLDME

>PvSR12

MAMPKGAFLTLVLLLLWASPWDGGFPPLERSSPHGCPQVHPSEGGSCTWGMWPHRFIPPSRIKGRTKASRLYAEGKVIYGLKEDKDFSVYAHFCYSRGRWEKGKKGVFIVTNFYIPNAKLLILEQTEQEMHSYVDGKNGNTCADLEKKALFLHPFDDIPPGDLKDSYFVYEGDVGDHLKGTQLSFVLLACGRQVKNAYKIEFRNNTHFLRNHFSCEEQGLIEIHLLLVVILGVLSLAYRAKQESLSRAHGALKEALHMAVIFFALSNICYLIHIFSFAFNGIGFTSLKVFSQINESIYDCLMLTIIFYIMGCTIDKERRREDTFRTSLTYGVLKFLYLLVEMQNQQELNLYASAVVALPFVAYRVVTAAMIYRNCKRLLMEKTPGEETFFILLQMFLYNLWILAIPAYYLLMSQASVHFTHLYVHFSNLLILIYLVHVVSEKKYEAMESRHPYLDMK

>PcSR12

MNKLCQRKKLLVFLFFLLYISNLGNQNLLQYEDSHIIEHNERGKYSYSIFSPLVEYIKGSNKIEERKSFSFFVSSKVIYGLYHNKNYSKFSDFCFIKKNNEKKGSVMLSNSYSPTTKLLVLDKTDNEIYNYTQNKNGKKCEDLEKEALYVYRFSDTPQENINNNYVFYNKDIDHQLENKSLNFIILHCDTKFKNAFKIEFVNNDNFLRNHFSCEEQGLIEIYMLLFVISTVLSLVYVRKRSMLNNANGALKESVHFGVLFFYFSNIFYLIHIYSYAFNGTGFSILKVLSQIYESIFDCITLTIIFYIVNTINNKKRRKEDTIKTGFIYSILKFFYILFEMQNHQSLNVYSSLHSVVALPFVSHRVIISVLIYNNCKKLLKEKTSASDKTRLLLDASMYIAWILSIPFIYFFLWNASMHFTHLFIHFSNLCILICLVYNISEKKYNSLESNHPYIDME

>PySR12

MSKFYQRKKVFVFLFLLLYISNWCNHILLQYKDSYIIEYNEKGKYMYLILFWLVEYINGSNKILERKPFSFFVSSKVVYGLYNNKNYSKFSDFCFIKKNNEKKGSVILSSAYSPTTKLLILDKTDDEIYNYTENKNGKKCEDLEKEALFVYRFSDMPQEYINKNYIFYNKDIDNQLENKLLNFIILHCDTKFKNAFKIEFVNNDNFLRNHFSCEEQGLIEISMLLFVILSVLSLIYFRKRNMLSKENGTLKESVHSGALFFYFSNIFYLIHIYAYALNGAGFSVLKVLSQIYESIFDCIILTILFYLINSIHNKKKRKEDTIKTGFIYSMLKFFYILFEIQNHQTLNAYSSLHSVVAFPFVSHRVVISVLIYNNCRKLLKEKTNIADKTNALLDASIYIAWILSIPFIYFFLWNASVHFTHLFIHFSNLSILICLVYKISEKKYNSLESNHPYIDME

>PfSR25

MAKRHKLKITILSIFFFVIFTGIHTVFTAFNRKDWLKFYTSCFGTGEVKWELLALLTVVNMLLLLLNVNYKENINHLNNKKSETSDINDDLINVDMHEFSNNEKDESSDENEKEKKYNKLKIRYLYNVSNSIICYYSLWILCYYLIYFLCFLSFLYGIRKFNNNVINIYTLRTCKIDKLTNYILSENTFISLYWAIINFNVFMSKYTDSFYVVNYFKLNFEFSNRKKKTLFILNYMYQLLLLSYTIYKNITLYTKGEYNLNQIICALIFLCLILYTILEITYVLEINRPCYNVQTKLPFHYVWAIIYLFIIFTSSVIFYFSVFSYSIKDQFVNFQITLWLFFISLTYIKKNQLFIKI

>PkSR25

MAKRHKLKITILPIFFFVIFTGIHTVFTAFDRNDWLMFYTSCSGSGQVKWELLGVLTILNSLILLLNVNYKENINHLNNKKSETSDINDDLINVDMNEFSNNEKDESSDESEKEKKYNKIKIRYLYSISNSRVCYYSMWILCYYLIYFLCFLSFLYGIRLFQNNLINIYTIRTCRIDNLANYILSENTFISLYWAIINFNVFMSKYTDSFYISNYFKLNIQFSTGKKKLLFFLNYAYQILLVSYTIYKNVDLYNKGLYNLNQIVCALIFLCLILYTILEITYVLEINKPCYYGVTKLSFNYIWAIIYLFVIFISSVIFYFSVFPYSIKDQYVNFQIMLWFFFISLTYIKRNQLFIKV

>PvSR25

MAKRHKLKITILPIFFFVIFTGIHTVFTAFDRNDWLKFYTSCSGTGQVKWELLGVLTILNSLILLLNVNYKENINHLNNKKSETSDINDDLINVDMNEFSNNEKDESSDESEREKKYNKIKIRYLYSISNSRVCYYSMWILCYYLIYFLCFLSFLYGIRLFNNNLINIYAIRTCKIDNLANYILSENTFISLYWAIINFNVFMSKYTDSFYISNYIKLNLEFTTGKKKLFFFVNYAYQILLLSYTIYKNVSLYNKGLYNLNQIVCALIFLCLILYTILEITYVLEINKPCYYGVTKLSFNYIWAIIYLFVIFISSVIFYFSVFSYSIKDQFVNFQIMLWFFFISLTYIKRNQLFIKV

>PbSR25

MAKRHKLKITTLSIFFFVILTGIHTVFTIFNQNDWIKFYTSCSGEGDVKWELLYVLTILNSLILIININYKENIDKLNNKKTEISDINDDLINVDINEFSNNERDDSDDELEKEKRYNKLKIKNLYSISNSRICYYSMWILCYYFIYFLCFLSFLYGIRLFNNNLINIYTIRTCKIEKLENYIISENTFTSLYWVFINFNVFMSKYTDSFYAINYVNFNIEFSTKKKRTLFFLNYAYQLLLITYSIYKNVLLYKRGLYNLNQIVCSIIFLCLILYTIFEITYVLEINKPSYYSMPKLSYNYVWSIIYLFVIFISSVIFYFSVYSYSIKDTFVNFQTMLWLFFISLTYIKRKQLFITP

>PySR25

MAKRHKLKITTLSIFFFVILTGIHTVFTTFNQKDWIKFYTSCSGEGDVKWELLYVLTILNSLLLMININYKENIDKLNNKKTEISDINDDLINVDINEFGNNERDDSGDELEKEKRYNKLKIKNLYSISNSRICYYSMWTLCYYCIYFLCFLSFLYGIRLFNNNLINIYTIRTCKIEKLENYIISENTFTSLYWVFINFNVFMSKYTDSFYAINYVNFNIEFSTKRKRTLFFLNYAYQLLLITYSIYKNVLLYKKGLYNLNQIVCSLIFLCLILYTIFEITYVLEINKPSYYSMPKLSYNYIWSIIYLFVIFISSVIFYFSVYAYSIKDTFVNFQIMLWLFFISLTYIKRKQLFITP

>PcSR25

MAKRHKLKITTLSIFFFVILTGIHTVFTTFNQKDWIKFYTSCSGDGDVKWELLYVLTLLNIFILMLNVNYKENIDTLNNKKTEISDINDDLINVDINEFGNNERDDSGDELEKEKRYNKLKIKTLYSITNSRICYYSMWILCYYSIYFLCFLSFLHGIRLFNNNLINIYTIRTCKLEKLNNYIISENTFTSLYWVFINFSVFMSKYTDSFYAINYVNFNIEFSTKRKRTLFFLNYAFQLLLITYTIYKNVLLYQNGVYNLNQIVCSLIFLCLILYTIFEIAYVLEINKPSYYSMPKLPYNYVWSIIYLFVIFVSSVIFYFSVYAYSIKDTFVNFQIMLWLFFLSLTYIKRKQLFIKA
