# Supplementary material for: Genome-Wide Detection of Serpentine Receptor-Like Proteins in Malaria Parasites
Source: PLoS One. 2008 Mar 26;3(3):e1889. doi: 10.1371/journal.pone.0001889 (PMC2268965; doi:10.1371/journal.pone.0001889)
Supplement: Data S3 — Alignments of the four putative Plasmodium serpentine receptor-like proteins highlighting transmembrane domains predicted by TMHMM 2.0, signal peptides and potential cleavage sites predicted by SignalP 3.0, and sites of probable N-glycosylation and phosphorylation as predicted by NetNGlyc 1.0 and NetPhos 2.0 Servers, respectively (0.20 MB DOC) [file pone.0001889.s003.doc]

**Supplementary data S3:** Aligments of the four putative *Plasmodium* serpentine receptor-like proteins highlighting transmembrane domains predicted by TMHMM 2.0, signal peptides and potential cleavage sites predicted by SignalP 3.0, and sites of probable N-glycosylation and phosphorylation as predicted by NetNGlyc 1.0 and NetPhos 2.0 Servers, respectively.

TM: transmembrane domain predicted by TMHMM 2.0^a^

i#: intracellular loop number

e#: extracellular loop number

SP: signal peptide (predicted by SignalP 3.0^b^)

Blue arrow indicate the potential signal peptide cleavage site.

Residues with potential to be phosphorylated are colored in pink (according to NetPhos 2.0 Server^c^).

N-Glyc: potential site for N-acetyl glycosylation (according to NetNGlyc 1.0 Server^d^)

^a^ http://www.cbs.dtu.dk/services/TMHMM/

^b^ http://www.cbs.dtu.dk/services/SignalP/

^c^ http://www.cbs.dtu.dk/services/NetPhos/

^d^ http://www.cbs.dtu.dk/services/NetNGlyc/

PvSR1 **MIKFAVGIICYYIVYGSYHVYQNVRTP**V**YDHSRD**G**KE**SG**K**E**Q**--------**EG**P**HVG**------**EKYIHRPFRNF**F**KQKDKVDYHLYMSCEEDIDLNRYVNEK**E**KYL**K**KNKN**

PkSR1 **MIKFAVGIICYYIVY**CA**YHVYQNVRTPIYEYAKDVKKNEKDQ**--------**EG*L*HVG**------**EKYIHR**A**F**T**NFLKQKDKVDYHLYMSCEE**N**IDLNRYVSEKDK**F**LEKNKN**

PfSR1 **MIK**III**G**V**I**G**YYI**L**YSSYH**I**Y**E**N**IKS**PI**FND**G**NV**IK**N**NE**G**D**KNEQLVIPK**EG*K***KRNSINEEE**E**E**YI**NK**PFKN**V**LK**KD**D**II**DYHLY**F**SCEEDID**I**N**KHMQ--**DKYLEK**DER

PvSR1 **FINVYNFNNVSYDW**S--**YSHPA**G**G**GG**NKWWN**V**LSPRKYPSV**K**LTIPRKLIKKNKEIYLHI**I**TYVNGELYRHGFVTS**A**LTR**G**KRGGKK**W**T**R**KEKYLWER**F**LDEEEDDEEEE**

PkSR1 **FINV**H**NFNNISYDWN**--**YSDPVEG**-**ETKWWNFLSPRKY**R**SVEVTIP**SS**LIKKNKEIYLHILTYVNGELYRHGFVTSILTREK**V**GAKKSTAKEKYLWERLL**E**EEE**------

PfSR1 **FI**S**VY**KIL**N**GK**Y**S**WN**NNVDLL**DE**KR**K**KNSFF**FL**FEKVH**PS**F**DI**S**IPKELI**DQR**K**D**IYLHILTYVN**K**ELYR**Y**G**SR**T**V**V**I**T**K**RKE**-KTT**T**R**GK**K**K**F**LW**KS**L**I**DEEEEEEEEE**

PvSR1 **EEEEEEEEEDDE**V**DE**A**YN**GQYGGHYTGEYNEQHAEQHA**DD**A**EGH**N**EAEEGDDDDD**D**EEEEEK*E***AE**GENDEETQTGDAS**-**N**EG**QKKSSGVATHEGVKKKN**K**KMKAKKR**---

PkSR1 -------**E**M**DD**-----**YN**----------------------**D**D**G**N**D**D**MEESEDD**N**DND**D**EDE**-----**ENEQKTQTDDSS**-**NDRQRKSVS**KQ**TQEGVKKKNNKMKAKKRPVK**

PfSR1 **EEEEEEEEE**EE**E**E**EE**EEE-------------------E**EEEE**E**EEE**E**EE**KKYQ**H**G**N**Y**E**KGG**S*Y***KV**H**K**N**I**S**NNKP**N**NKNP**NDK**RDIKHN**NN**N**N**NNKM**KK**D**N**FI**K**SNQN**SLN**

PvSR1 **H**A**KK**T**K**Q**K**-----**IFYIPKKVKFGPVIEHNDINVNKIGFFSNIFLDKENSVYLLPTYYNDHLT**A**EDEYELLQIG**-------------**GEGEKEDVG**K**KKKKKN**-**AQEDN**-

PkSR1 **KKKKKKKK**-----N**FYIPKKVKFGPVIEHNDINVNKIGFFSNIFLD**T**ENSVYLLPTYYNDHLTPEDEYELLQIV**-------------**WEGEKEDVIA**R**KRKKNNTQHD**H-

PfSR1 I**HKKKKK**RKHKDF**LFYIPKK**IR**FGPVIE**Y**ND**FHIS**K**L**GFFSN**MHV**DK**DTNT**YLLP**I**Y**I**N**ND**LTP**D**DEY**RMIKMKNSDDMMKKKLKKKRSS**EK**N**D**P**YYK**ENVR**NDN**NNN**N**N

**TM-I**

PvSR1 -------------------**EM**Y**S**K**DN**------------------**HTQMGN**V**IEIEYSPISLPQFNLYNIIIFNVNYAKEKYK**IA**AYDLDSLVIHFCGNISLCLII**CL**LCL**

PkSR1 -------------------VT**KSDDN**------------------Y**TH**R**GNIIEIE**L**SPISLPQFNLYNIILFN**I**NYAKEKYKFV**T**YDLD**N**LIIHFCGNI**Y**LCLIIFILC**F

PfSR1 NNNIKNNYLEIYDLSKRTY**EMR**N**E**R**N**KKIYEKNSSINNNVHKLE**H**EL**M**DY**II**N**IEY**V**PI**NYNYY**NL**L**N**MLK**FNV**S**Y**V**K**K**KY**N**FIS**F**D**M**DS**ITTFL**C**CH**IT**CSM**IIYILCI**

PvSR1 **ILLL**I**DLVAL**V**FDLTSWNRLNGLYSFSS**-**DALHFK**F**LFSMFIFLYLKNKSNCKILMVFCVAKMAVCLWKLLDRYDIEFMEVHPYVCIT**S**ST**GG**GA**GSAGSARSGVGGGSG

PkSR1 **ILLLMDL**M**ALLFD**W**SRWNRVNDLYSFPS**-Y**TVHFKLIFTLFIFLYLKNKNSC**N**ILMVFCVAKMAVCLWKLLDHYDIEFMEIHPYV**R**ITRNI**EE**IS**---------------

PfSR1 **I**YII**M**EI**T**Y**LLFDI**KM**W**K**R**W**NNLY**T**FT**YN**NDI**VMN**I**TLL**FFI**L**LYL**R**N**I**D**YG**R**VI**M**IYYIM**KM**M**V**LIF**K**IIYN**YDI**CILNDY**PY**I**C**MN**K**---------------------

**TM-II**

**TM-III**

**TM-IV**

**TM-V**

**TM-VI**

**TM-VI**

**TM-VII**

**i1**

**e1**

**i2**

**i2**

**e2**

**i3**

PvSR1 ND**N**P**GN**G**GGSGSG**S**G**G**GA**G**THK**R**QSRSEVEDLERYIKMKMPNVMICTVVSTCAYNFMYTQYDSVYAFIIHSVAVCSYIFNFLFMCPQIVRNY**H**T**K**TVERVPLFFFFFLFL**

PkSR1 --**N**I**GE**R**GNENQS**I**D**K**GAI**IQ**KNQ**F**RTELE**N**IE**T**YIKMKMPNVM**F**CTVVSMCAYNFIYTQY**E**SIYAFIIHSIAICSYIFNFVFMCPQIVRNYYTRTVERVPLFF**L**FFLFL**

PfSR1 ----------------KS**LKE**M**N**K**E**MIMD**EE**F**EK**K**IK**K**K**VNIF**MI**FSIIL**I**FI**YN**Y**FYT**K**YDS**Y**Y**SYV**IH**T**L**GFS**SY**LYK**FI**L**M**L**PQI**IT**N**I**YTRTV**Q**R**MSFP**FF**L**FL**LV

**e3**

PvSR1 **YA**I**MDDLF**A**LVLRMPLVHKWNALGDDLVFFIFLLQYCVYKKGDSRV**AP**GEAPAAP**P**G**T**KAQRECKKRK**

PkSR1 **YALMDDLFVLVLR**I**PLVHKWNALGDDIVFFIF**F**VQYCVYKKGKSRVGSGEVAAAPQGAKPQRESKKKK**

PfSR1 NV**L**IN**DLFI**IF**LRMP**K**VHK**YYLFA**DD**FIL**F**L**FIIQYC**I**YKK**ENKIF**GA**R**E**KLVLL**K**N**A**------**KK**N**K**

PySR10 **MVIWKAN**P**KNKN**---**LLYLL**F**LYIFFISFTNCQLIKLDGQKINTNYILYVLKGLYIYGK**D**DAPYILLGEKKDMNN**AI**PHAIFENVGISTNE**I**KNTKYFTFG*R*KN**------

PbSR10 **MVIWKAN**P**KNKN**---**LLYLL**F**LYIFFISFTNCQLIKLDGQKINTNYILYVLKGLYIYGKNDVPYILLGEKKDMNS**A**GPHAIFENVGISTTE**I**KNTKYFTFG*R*KN**------

**TM-I**

**TM-II**

**TM-III**

**e1**

**TM-IV**

**i2**

**TM-V**

**e2**

**TM-VI**

**i3**

**TM-VII**

# SP

PcSR10 **MGIWKAN**P**KNKN**---**LLYLL**F**LYIFFISFTNCQLIKLDGQKINTNYILYVLKGLYIYGKNDAPYILLGEKKDMNN**A**GPHAIFENVGISTNE**I**KNTKYFSFG*M*KH**------

PkSR10 **M**-**IWKST**Q**KVKC**KTE**FLIVV**-**FLVLFASITNSQLIKLDGQKI**S**TNYILYVLKGLYIFGKNDTPYVLLGEKKDMAT**K**GPHAIFENIGISTTD**N**KNTKYFSFD*I*EE**SRTNGQ

PvSR10 **MMIWKST**Q**KVKC**KSE**FLIVV**-**FLLLFASIT**S**SQLIKLDGQKINTNYILYVLKGLYIFGKNDSPYVLLGEKKDMAT**K**GPHAIFENIGISTTD**N**KNTKYFSFD*M*EE**------

PfSR10 **MVIWKG**--N**VKN**KIL**FLI**F**V**A**YF**F**VFV**K**I**S**NGQLIKLDGQKINTNYILYVLKGLYIFG**E**N**E**SPYVLLG**K**KKDMD**FK**A**A**HAIFENVGISTTD**N**KNTKYFSF**E***M***G**D**TTSGNN

PySR10 ---------------**NSDHDDN**V**NEKND**EDGE-------------MEKV**NNNNSTS**Y**EE**-**DDESEEKKKKK**G**FKLNLYKDNPYVQRKTEH**S**D**L**ED**L**DSN**D**NTSDLFLEII**

PbSR10 ---------------**NSDHNDN**A**NEKND**EDGE-------------MEKT**NNNNSTS**Y**EEEDDESEEKRKKK**G**FKLNLYKDNPYVQRKTEH**S**D**L**ED**W**DSN**D**N**I**SDLFLEII**

PcSR10 ---------------D**S**H**HDDN**I**NEKHN**EDGE-------------AEKAK**NHN**H**NS**Y**DDEDDESDE**NN**KKK**G**FKLNLYKDNPYVQRKTEH**S**E**T**K**SWN**SN**D**NTSDLFLEII**

PkSR10 EN-------------**NESDEEN**K**SDEHP**---------------------**TSE**M**N**H**S**S**D**G**DDDDDDEKDKKD**K**FKINLYKDNPYLRKKKEY**M**H**K**HE**D**DEV**L**DAGNLFLEVI**

PvSR10 ---------------**NKSDEE**HK**ADEHS**---------------------**TSESNNG**S**DDEDEDSDEKDKKD**K**FKINLYKDNPYLRKKKEY**M**H**K**HE**E**DEV**L**NTGNLFLEV**V

PfSR10 ENNNNNNNDGHNNNN**NDSHN**N**N**N**ND**G**HN**NNYDHNNDSTLENTNLPQNSY**NNNGNNG**NN**SS**EKHK**DEDE**D**KD**K**FKINLYKDNPYLRKKKEY**RYS**ED**V**DS**FV**TS**-E**LFLELI**

PySR10 **IMKESDFNKLYLPKDSNMCCYTE*M*TGVDNNDKYTCPGKGYLKRYLDESEMHSLKVPIYFINDRIEDD**-----------------------------**NTSS**GN**EVNHNKFL**

PbSR10 **IMKESDFNKLYLPKDSNMCCYTE*M*TGIDNNDKYTCPGKGYLKRYL**G**ESE**F**HSLKVPIYFINDRIEDD**-----------------------------**NTSS**GN**EVN**Y**NKFL**

PcSR10 **IMKETDFNKLYLPKDT**KR**CCYTK*M*TGFDNSDKYTCPGKGYLKRYLDESEMHSLKVPIYFINDRIEDN**-----------------------------**DTSS**GN**EVNHNKFL**

PkSR10 **IMKESDFNKFYLPKDTNVCCHME*E*NGMDGNDSYTCPGRGYLKRYVEESSMYSLKLPVYFLNDRISND**-------------------------DEIS**PLEN**--**EVNHENFL**

PvSR10 **IMKESDFNKFYLPKDSNVCCHTE*E*RGMDGND**A**YTCPGRGYLKRYVKESSMY**A**LKLPVYFLNDRISNN**-------------------------DGMS**PLEN**--**EVNHEEFL**

PfSR10 **IMKE**K**DFNKHYLPKD**H**DICCYM**Q***E*EGIDG**YE**KYTCPGKGYLKRYVDE**E**HMYSLKLPVYFINDRIKDD**TNNNNNNNNNNNNNNSSSSSSSSYYNNMY**NLNN**GN**E**I**NHEN**LI

**i1**

PySR10 **E*L*IKNEHIYNIDKTDIYTIFLSNCGDSK*I*YELDLHGNIHILNKYGYLPGDKITKLNLYV**S**LMLIYFIY**S**IIW**S**Y**S**LIKNK**AN**VIKIQVWI**S**VCIFLYLLENLFLYLYFMT**

PbSR10 **E*L*IKNEHIYNIDKTDIYTVFLSNCGDSK**V**YELDLHGNIHILNKYGYLPGDKIPKLNLYV**S**LMIIYFIY**S**MIW**S**Y**S**LIKNKT**N**VIKIQVWI**S**VCIFLYLLENMFLYLYFMT**

PcSR10 **E*L*IKNEHV**F**NIDKTDIYTVF**I**SNCGDSK*I*YELELHGNIHILNKYGYLPGDKITKLNLYV**S**LMIIYL**L**Y**S**IIW**S**Y**S**LFKNKT**N**VIKIQVWI**S**VCM**L**LYLIENIFLYLYFMT**

PkSR10 **K*K*IQNKHIYNIDDTDVYALFLSNCSDSK*K*YELELHGNIHILNKYGYLPGDKIPKLNLYV**V**CMLIYAIY**L**FTW**I**Y**L**LIRNKQ**F**VIKIQIWI**L**VCTFLYL**M**ENVFLFLYFLV**

PvSR10 **K*K*IQ**S**RHVYNIDETDVYALFLSNCSDSK*K*YELELHGNIHILNKYGYLPGDKIPKLNLYV**L**CMIIYAIY**L**FAW**I**Y**L**LMRNKQ**F**VIKIQIWI**L**VCIFLYLLENFFLFLYFLV**

PfSR10 N***H***L**KNK**F**VYNI**K**DTDVYALFLSNC**L**DSK*K*YEL**H**LHGNIHILN**D**YGYLPGDKISKLNLYV**LS**MIIY**S**IY**L**FIW**S**Y**L**LIRNKN**Y**VIKIQIWI**L**VCVFLYLIENI**C**LFLYFLS**

**i3**

PySR10 **YNVQAKINNNYLFMAVF**F**SVLKNVCSYLLILLGSLGWGLVIPTLDRKTFIKIKVLFIFFIIFDFIKQFLDAHLAD**E**HVNTVYFLCCILPMSIIYAIIYVWIFISSSKIII**

PbSR10 **YNVQAKINNNYLFMAVF**F**SVLKNVCSYLLILLGSLGWGLVIPTLDRKTFIKIKVLFIFFIIFDFIKQFLDAHLAE**E**HVNAVYFLCCILPMSIIYAIIYIWIFISSSKIII**

PcSR10 **YNVQAKINNNYLFMAVF**F**SVLKNVCSYLLILLGSLGWGLVIPTLDKKTFIKIKVLFIFFIIFDFIKQ**L**LDAHLAE**E**HVNTVYFLCCILPMSIIYSIIY**M**WVFISSSKIII**

PkSR10 **YNLRARVNSNLLFLSVC**S**SILKNVCSYLLILLGSLGWGLVIPTLDKKTFIKIKVLFFFFIIFDFIKQFLDMHLTD**A**EVNAVYFLFCIIPVTIIYSIIYLWVFTSASKIII**

PvSR10 **YNIRARVNSNLLFLSVC**T**SILKNVCSYLLILLGSLGWGLVIPTLDKKTFIKIKVLFFFFIIFDFIKQFLDMHLTD**A**EVNAVYFLFCIIPVTIIYSIIYLWVFTSASKIII**

PfSR10 **YNL**Y**AKVNN**E**LLFISVC**S**SILKNVCSYLLILLGSLGWG**I**VIPTLDRKTFIKIK**I**LFFFFIIFDFIKQF**V**DMHLTD**TQI**NT**G**YF**F**FCIIPVTIIYSIIYIWVFTSAS**Q**III**

**e3**

PySR10 **QLNEDKQYEKLNMFKNFFNVLILALIFSIISLIIDLFVMLFPSDQLWNLKCYISEGV**N**SFLFLTVLTAMCMLFKPSERLKRISHFTEIGDMDEMDDFSHFKNSIEDIS**

PbSR10 **QLNEDKQYEKLNMFKNFFNVLILALIFSIISLIIDLFVMLFPSDQLWNLKCYISEGV**N**SFLFLTVL**S**AMCMLFKPSERLKRISHFTEIGDMDEMDDFSHFKNSIEDIS**

PcSR10 **QLNEDKQYEKLNMFKNFFNVLILALIFSIISLIIDLFVM**M**FPN**E**QLWNLKCYISEGV**N**SCLFLTVLTAMCVLFKPSERLKRISHFTEIGDMDEMDDFSHFKNSIED**V-

PkSR10 **QLNEDKQYEKLNMFKKFFNVLIFSLIFSVISFVIDIVVMLFVDNTIWSLKCYISEGI**I**SCLFLIIITAMFMLFRPSDRLKRISHFTEIGDMDEMEDFSQFKGSIEDIS**

PvSR10 **QLNEDKQYEKLNMFKKFFNVLIFSLIFSVIAFVIDIVVMLFVDNTIWSLKCYLSEGI**I**SCLFLIIITAMFMLFRPSDRLKRISHFTEIGDMDEMEDFSHFKAS**M**EDIS**

PfSR10 **QLNEDKQYEKLNMFKN**L**FNVLIF**T**L**L**FSVIAFIIDIVVML**Y**VDNSIWNLK**N**YLSEGI**I**SCLFLIILTAMFILFKPSDRLKRISHFTEIGDMDEMEDFSNFKNSIEDIS**

**TM-I**

**i1**

**TM-II**

**TM-III**

**TM-IV**

**i2**

**TM-V**

**TM-VI**

**TM-VI**

**TM-VII**

**e2**

# N-Glyc

# N-Glyc

# N-Glyc

PcSR12 -**MNK*L*CQR*K*KLLVFLFFLLYISNLGNQ**N**LL*QY*EDSH**---**IIEHNER**-----G**KY**-S**Y**S**IF**SP**LVEYI**K**G**S**NKI*E*ERK**S**FS**-------**FFVSSKVIYGLYHNKNYSKFSDF**

PySR12 -**MSK*F*YQR*K*KVFVFLFLLLYISNWCNH**I**LL*QY*KDSY**---**IIEYNEK**-----G**KY**-M**Y**L**IL**FW**LVEYI**N**G**S**NKI*L*ERK**P**FS**-------**FFVSSKV**V**YGLYNNKNYSKFSDF**

PfSR12 M**IRR*K*WSK*I*KLAIYFIAFYYLTKIDEK**C**LL*IK*EGEL**NLS**ITNVHDN**NYMISE**KY**NK**Y**Y**IL**SL**LYKFG**G**I**L**KEI*Y*D**K**K**I**F**VKNKSCNN**F**M**VSSKVIYGLYND**M**NYSKYS**N**F**

PkSR12 ----------------------------------------------------------------------MNV***K***G**R**SIA**S**G------**FY**ATG**KVIYGL**K**E**E**RN**F**S**V**Y**G**DF**

PcSR12 **CF**I**K**--**KNN**E**KKGSV**M**LSNSYSPTTKLL**V**LDKTD**N**EIYNYTQNKNGKKCEDLEKEAL**Y**VY**R**FSDTPQE**N**INNNYVFYNKDIDHQLENK**S**LNFIILHCDTKFKNAFKIEF**V

PySR12 **CF**I**K**--**KNN**E**KKGSVILSSAYSPTTKLLILDKTDDEIYNYTENKNGKKCEDLEKEALFVY**R**FSDMPQE**Y**INKNYIFYNKDID**N**QLENK**L**LNFIILHCDTKFKNAFKIEF**V

PfSR12 **CF**S**K**--N**AN**--**NGVVILSN**L**YVPNTK**F**LILDKSDDEIYNYGKNKNGK**T**CEDLEK**I**ALFVH**PL**NDIP**P**E**LM**NKTYFVYQKDIEKSL**T**DK**K**LNFILLNCGNKIKNAFKIEF**K

PkSR12 **C**YSRSR**HT**MG**RNGV**L**I**VT**SSY**I**PNTKLLIL**K**KV**E**E**D**I**N**NY**M**N**G**KNG**N**RC**A**DLE**R**KALFVH**P**FD**N**TPE**GN**L**P**NSY**S**LYE**E**DI**G**DNLKD**TP**LNF**V**LL**A**CG**RH**VKNA**Y**KIEF**R

**e1**

PcSR12 **NNDNFLRNHFSCEEQGLIEIYMLLFVI**S**TVLSLVYVRKRS**M**LNNANGALKESVHFGVLFFYFSNIFYLIHIYSYAFNGTGFSILKVLSQIYESIFDCI**T**LTIIFYIVNTI**

PySR12 **NNDNFLRNHFSCEEQGLIEI**S**MLLFVILSVLSLIYFRKRN**M**LS**KE**NGTLKESVH**S**GALFFYFSNIFYLIHIYAYA**L**NGAGFSVLKVLSQIYESIFDCIILTI**L**FY**L**INSI**

PfSR12 **NN**M**NFL**K**NHFSCEEQGL**F**EIHMLLIV**L**L**F**VLSLVYYRKR**KN**LNNTN**N**VLKEAIHCS**Y**LFFLLSNILY**F**IH**LI**SYAFNGSGFSILKVLSQIYE**A**IFDCFIL**V**II**Y**YIFN**--

PkSR12 **NNA**H**FLRNHFSCE**D**QGLIEIH**F**LLVVILVVLSL**A**Y**KSRQ**D**S**L**R**GA**H**SA**M**KEGIHMSV**M**FFVLSN**L**CYLIHIF**F**YAF**D**GTG**LTS**LKVLSQ**MG**ESI**Y**DCFV**M**TIIFYIM**C**CT**

**i3**

PcSR12 **NNKK**R**RKEDTIKTGFIYSILKFFYILFEMQNHQSLNVYSSLHS**----------------------------**VVALPFVSHRVIISVLIYNN**C**KKLL*K*EKTS**A**SDKT*R*LLL**

PySR12 **HNKKKRKEDTIKTGFIYS**M**LKFFYILFEIQNHQTLN**A**YSSLHS**----------------------------**VVA**F**PFVSHRV**FYMK**LFY**------------------**LVL**

PfSR12 **ND**MQ**K**K**KE**E**TIR**V**AFTYSILKFIYILFEIQNNQ**E**L**D**LYS**T**LHS**----------------------------**IVALPFVVYR**I**IVAVL**N**YDN**S**KKLL*K*EKTQ**V**DEKF*Y*VL**F

PkSR12 M**DREKR**RK**DT**F**RTA**LN**Y**G**VLKFLY**L**L**V**EMQNQ**E**DLNLY**A**SLHS**GWGGLANYGTVTSRVEEMPTPSYSLPLS**LVALPFV**L**YRVIIAA**T**IYRN**Y**KRLL*M*EKTS**R**EETF*F*I**S**L**

**e3**

PcSR12 **DA**S**MYEP*KK***KK**AM**S**QWGV*Y*RM**D**SLYSVY**L**FFFV*E*CF**NALYP***F*V*H*SF**L***Q***PMH***I***N**M*F*G**I**Q**H**F**REEV

PySR12 F**GFIFEG**----**LVYKIS**E***K*KY**N**SL**E**SNH**P**YI**D**M*E***------------------------------

PfSR12 **DTFFYN**L***WI***LS**I**P**VQY**F**L*M*K**SF**SLH**F**TH**L**FVHF*F*NL**YILIY***L*V*Y*NL**S***E***EKF***E***V**L*E*S**K**H**P**Y**LDLN

PkSR12 **H**M**FL**LV**N*EE***GYN**LW**I**LSI**PA**Y**YL**LMS**RASI**FGA*Y*HF**G---E***E*V*R*SH**G***I***QAS***L***L**G*Y*G**V**T**P**F**A---

PbSR25 **MAKRHKLKITTLSIFFFVILTGIHTVFT**I**FNQNDWIKFYTSCSGEGDVKWELLYVLTILN**S**LILIININYKENIDKLNNKKTEISDINDDLINVDINEFSNNERDDS**D**DE**

PySR25 **MAKRHKLKITTLSIFFFVILTGIHTVFTTFNQKDWIKFYTSCSGEGDVKWELLYVLTILN**S**LLLMININYKENIDKLNNKKTEISDINDDLINVDINEFGNNERDDSGDE**

PcSR25 **MAKRHKLKITTLSIFFFVILTGIHTVFTTFNQKDWIKFYTSCSGDGDVKWELLYVLTLLN**IF**ILMLNVNYKENID**T**LNNKKTEISDINDDLINVDINEFGNNERDDSGDE**

PkSR25 **MAKRHKLKITILPIFFFVIFTGIHTVFTAFDRNDWL**M**FYTSCSGSGQVKWELLGVLTILN**S**LILLLNVNYKENINHLNNKKSETSDINDDLINVDMNEFSNNEKDESSDE**

PvSR25 **MAKRHKLKITILPIFFFVIFTGIHTVFTAFDRNDWLKFYTSCSGTGQVKWELLGVLTILN**S**LILLLNVNYKENINHLNNKKSETSDINDDLINVDMNEFSNNEKDESSDE**

PfSR25 **MAKRHKLKITILSIFFFVIFTGIHTVFTAFNRKDWLKFYTSC**F**GTGEVKWELLA**L**LTV**V**N**M**LLLLLNVNYKENINHLNNKKSETSDINDDLINVDM**H**EFSNNEKDESSDE**

PbSR25 L**EKEKRYNKLKIK**N**LYSISNSRICYYSMWILCYYFIYFLCFLSFLYGIRLFNNNLINIYTIRTCKIEKL**E**NYIISENTFTSLYWVFINFNVFMSKYTDSFYA**I**NYVNFNI**

PySR25 L**EKEKRYNKLKIK**N**LYSISNSRICYYSMW**T**LCYYCIYFLCFLSFLYGIRLFNNNLINIYTIRTCKIEKL**E**NYIISENTFTSLYWVFINFNVFMSKYTDSFYA**I**NYVNFNI**

PcSR25 L**EKEKRYNKLKIK**T**LYSI**T**NSRICYYSMWILCYY**S**IYFLCFLSFL**H**GIRLFNNNLINIYTIRTCK**L**EKL**N**NYIISENTFTSLYWVFINF**S**VFMSKYTDSFYA**I**NYVNFNI**

PkSR25 S**EKEKKYNKIKIR**Y**LYSISNSRVCYYSMWILCYYLIYFLCFLSFLYGIRLF**Q**NNLINIYTIRTC**R**IDNL**A**NYILSENTFISLYWAIINFNVFMSKYTDSFYI**S**NYFKLNI**

PvSR25 S**E**R**EKKYNKIKIR**Y**LYSISNSRVCYYSMWILCYYLIYFLCFLSFLYGIRLFNNNLINIY**A**IRTCKIDNL**A**NYILSENTFISLYWAIINFNVFMSKYTDSFYI**S**NYIKLNL**

PfSR25 N**EKEKKYNKLKIR**Y**LY**NV**SNS**I**ICYYS**L**WILCYYLIYFLCFLSFLYGIR**K**FNNN**V**INIYT**L**RTCKIDKL**T**NYILSENTFISLYWAIINFNVFMSKYTDSFYV**V**NYFKLN**F

**TM-I**

**i1**

# SP

**TM-II**

**i1**

**TM-III**

**e1**

**i2**

**TM-IV**

**i2**

**TM-V**

**e2**

**TM-VI**

**i3**

**e3**

PbSR25 **EFST**K**KKRTLFFLNYAYQLLLITYSIYKNV**L**LYKRGLYNLNQIVCS**I**IFLCLILYTIFEITYVLEINKPSYYSMPKLSYNYVWSIIYLFVIFISSVIFYFSVYSYSIKDT**

PySR25 **EFST**K**RKRTLFFLNYAYQLLLITYSIYKNV**L**LYKKGLYNLNQIVCSLIFLCLILYTIFEITYVLEINKPSYYSMPKLSYNYIWSIIYLFVIFISSVIFYFSVYAYSIKDT**

PcSR25 **EFST**K**RKRTLFFLNYA**F**QLLLITYTIYKNV**L**LYQ**N**GVYNLNQIVCSLIFLCLILYTIFEI**A**YVLEINKPSYYSMPKLPYNYVWSIIYLFVIFVSSVIFYFSVYAYSIKDT**

PkSR25 Q**FST**G**KKKLLFFLNYAYQILLVSYTIYKNV**D**LYNKGLYNLNQIVCALIFLCLILYTILEITYVLEINKPCYYGVTKLSFNYIWAIIYLFVIFISSVIFYFSVF**P**YSIKDQ**

PvSR25 **EF**T**T**G**KKKL**F**FF**V**NYAYQILLLSYTIYKNV**S**LYNKGLYNLNQIVCALIFLCLILYTILEITYVLEINKPCYYGVTKLSFNYIWAIIYLFVIFISSVIFYFSVFSYSIKDQ**

PfSR25 **EFS**NR**KKKTLF**I**LNY**M**YQLLLLSYTIYKN**IT**LYTKG**E**YNLNQI**I**CALIFLCLILYTILEITYVLEIN**R**PCY**NVQ**TKLPF**H**YVWAIIYLF**I**IF**T**SSVIFYFSVFSYSIKDQ**

**TM-VII**

PbSR25 **FVNFQ**T**MLWLFFISLTYIKRKQLFITP**

PySR25 **FVNFQIMLWLFFISLTYIKRKQLFITP**

PcSR25 **FVNFQIMLWLFF**L**SLTYIKRKQLFIKA**

PkSR25 Y**VNFQIMLWFFFISLTYIKRNQLFIKV**

PvSR25 **FVNFQIMLWFFFISLTYIKRNQLFIKV**

PfSR25 **FVNFQI**T**LWLFFISLTYIK**K**NQLFIK**I
