# Supplementary material for: Genome-Wide Detection of Serpentine Receptor-Like Proteins in Malaria Parasites
Source: PLoS One. 2008 Mar 26;3(3):e1889. doi: 10.1371/journal.pone.0001889 (PMC2268965; doi:10.1371/journal.pone.0001889)
Supplement: Data S4 — Genomic sequences of four serpentine receptors of different species of malaria parasites. Sequences corresponding to introns are red-colored, and coding sequences are presented in black (0.07 MB DOC) [file pone.0001889.s004.doc]

**Supplementary data S4:** Genomic sequences of four serpentine receptors of different species of malaria parasites. Sequences corresponding to introns are red-colored, and coding sequences are presented in black.

**1) Genomic sequences *P. falciparum* serpentine receptor PfSR1 (PF11_0321) orthologs:**

>PfSR1(PF11_0321) ATGATTAAAATTATTATAGGTGTAATAGGTTATTATATTTTATACAGCTCCTACCATATATATGAAAATATTAAGAGTCCAATTTTTAATGATGGTAATGTTATAAAAAATAATGAAGGTGATAAAAACGAGCAATTGGTTATCCCTAAGGAGGGTAAAAAAAGGAATAGTATTAATGAAGAAGAAGAGGAATATATAAATAAGCCTTTTAAGAATGTATTAAAAAAAGATGACATAATTGATTATCATTTATATTTTTCTTGTGAAGAAGATATAGATATAAATAAACATATGCAAGACAAATATTTAGAAAAGGATGAAAGATTTATCAGTGTATATAAAATATTGAATGGTAAATATAGCTGGAATAATAATGTAGATTTGTTAGATGAAAAACGGAAGAAGAATTCTTTTTTTTTTTTGTTTGAAAAAGTACATCCATCATTTGATATTAGTATACCCAAAGAATTAATTGATCAAAGGAAGGATATTTATTTGCATATCTTGACTTATGTTAATAAGGAATTGTACAGGTATGGTTCAAGAACGGTTGTGATAACAAAGAGGAAGGAAAAGACAACAACACGAGGTAAGAAAAAGTTTTTGTGGAAAAGTCTTATTGATGAGGAGGAAGAAGAAGAGGAAGAGGAGGAGGAAGAAGAAGAGGAAGAGGAGGAGGAAGAAGAAGAGGAAGAGGAGGAGGAAGAGGAGGAAGAGGAGGAGGAAGAAGAAGAGGAAGAAAAAAAATATCAACATGGAAATTATGAAAAAGGAGGATCCTATAAGGTTCATAAAAATATATCAAACAATAAACCAAATAATAAAAATCCAAATGACAAAAGGGATATAAAACATAACAATAATAATAATAATAATAAAATGAAGAAGGATAATTTTATTAAAAGTAATCAAAACAGTTTGAATATTCATAAAAAAAAAAAGAAAAGAAAACATAAAGATTTTTTATTTTATATTCCCAAAAAAATAAGATTTGGACCGGTTATTGAATATAACGATTTCCATATTAGTAAGTTAGGTTTTTTTTCAAACATGCATGTAGATAAAGATACGAATACATATTTGTTACCTATATATATTAATAATGATTTAACACCTGATGATGAGTATCGAATGATTAAGATGAAAAATTCTGATGATATGATGAAAAAGAAACTAAAAAAGAAACGATCATCAGAAAAAAATGATCCATATTATAAAGAGAATGTAAGAAATGATAATAATAATAATAATAATAATAATAATATAAAGAATAATTATTTAGAAATTTACGACTTGTCAAAGAGGACATATGAAATGAGAAATGAGAGGAACAAGAAGATATACGAGAAAAATAGTAGCATAAATAATAATGTTCATAAATTAGAACATGAACTAATGGATTATATAATAAATATAGAATATGTGCCTATAAATTATAATTATTATAATTTGTTGAATATGTTAAAATTTAATGTATCATATGTAAAAAAGAAATATAATTTTATATCTTTTGATATGGATAGTATAACTACATTTTTATGTTGTCATATAACATGTAGCATGATAATATATATATTATGTATTATTTATATTATAATGGAAATTACGTATTTATTATTTGATATAAAAATGTGGAAACGTTGGAATAATTTATACACCTTTACATATAATAATGATATTGTTATGAATATCACATTACTTTTTTTTATCTTATTATATTTAAGGAATATTGATTATGGTAGAGTTATTATGATATATTATATAATGAAGATGATGGTACTAATATTTAAAATAATATATAATTATGATATATGTATATTGAATGATTATCCATATATATGTATGAATAAAAAGTCTTTAAAAGAAATGAATAAAGAAATGATAATGGATGAAGAATTTGAAAAAAAAATAAAAAAAAAAGTAAATATATTTATGATCTTTAGTATTATCCTAATATTTATATATAATTATTTTTATACAAAATATGATTCTTATTATTCATATGTAATACATACACTTGGTTTCTCATCCTATCTTTATAAATTTATTTTAATGTTACCTCAAATTATAACAAATATTTACACACGTACCGTACAGAGGATGTCATTTCCATTTTTTCTTTTCTTACTAGTGAATGTATTAATTAATGATCTTTTTATAATTTTTTTAAGAATGCCAAAGGTACATAAATATTATTTATTTGCAGATGATTTCATTCTCTTTCTATTTATAATACAATATTGTATTTATAAAAAAGAAAATAAAATATTCGGTGCTCGAGAAAAGTTGGTACTTTTGAAGAATGCAAAAAAAAATAAATGA

>Pk_279e09p1c|Plasmodium knowlesi

ATGATAAAGTTTGCAGTGGGAATAATATGCTACTACATTGTGTACTGTGCTTATCACGTGTATCAAAATGTAAGAACCCCCATATATGAATACGCAAAGGATGTGAAAAAAAACGAAAAGGACCAGGAAGGATTACACGTGGGGGAGAAATATATTCATAGAGCATTCACAAATTTCCTTAAACAAAAGGATAAAGTGGATTATCACCTGTATATGTCGTGTGAAGAAAATATTGACTTGAATAGATACGTAAGTGAGAAAGACAAATTTTTGGAAAAAAATAAAAATTTTATTAATGTGCATAATTTTAACAACATATCATATGATTGGAATTATAGTGATCCTGTAGAAGGAGAAACAAAGTGGTGGAACTTCCTGTCGCCGAGGAAGTATCGTTCCGTTGAGGTAACCATCCCAAGTAGTCTCATAAAGAAGAACAAAGAAATTTACCTCCACATACTTACCTACGTGAATGGAGAACTCTACAGGCATGGGTTCGTCACTAGTATACTGACGAGGGAAAAAGTCGGGGCAAAGAAGTCGACTGCCAAGGAGAAGTACCTCTGGGAGCGCCTCCTAGAAGAGGAAGAAGAAATGGACGACTATAACGATGATGGGAATGATGACATGGAAGAAAGTGAAGATGATAATGATAATGATGATGAGGACGAAGAAAATGAACAGAAGACACAGACAGATGACTCGTCAAACGATAGGCAAAGGAAAAGCGTGAGCAAACAGACCCAGGAAGGAGTAAAAAAAAAAAATAATAAAATGAAAGCAAAAAAAAGGCCAGTAAAAAAAAAAAAAAAAAAAAAAAAAAAAAATTTTTACATTCCCAAGAAAGTCAAATTTGGGCCTGTCATCGAGCATAATGATATTAACGTAAATAAAATTGGATTTTTCTCCAACATATTTCTGGATACAGAAAATAGTGTGTACCTTTTGCCAACCTACTACAATGATCATCTAACCCCGGAAGATGAATATGAACTATTGCAAATAGTATGGGAGGGAGAGAAGGAAGATGTAATTGCAAGGAAAAGAAAAAAAAATAACACACAACATGATCATGTGACCAAGTCGGATGACAACTATACGCATCGTGGAAATATTATCGAAATTGAGTTATCCCCAATTAGCCTCCCCCAGTTCAATCTGTACAACATTATCCTGTTCAACATAAATTACGCGAAAGAAAAATATAAATTTGTAACTTACGATTTGGATAACTTGATCATACACTTTTGTGGAAATATATATTTGTGCCTCATCATATTTATTCTTTGTTTCATCCTTTTGTTAATGGATCTCATGGCTCTATTATTTGATTGGTCTAGATGGAACAGGGTGAATGACTTATATTCCTTCCCATCCTATACCGTTCATTTTAAGTTAATCTTTACCTTGTTCATATTTCTTTATTTGAAAAATAAAAATAGTTGCAATATATTGATGGTGTTTTGCGTGGCGAAGATGGCCGTTTGCCTTTGGAAGTTACTGGACCATTACGACATTGAATTTATGGAAATTCATCCTTACGTGCGCATTACGAGGAATATCGAGGAGATCAGTAATATCGGCGAAAGGGGAAATGAAAATCAAAGCATCGACAAGGGTGCCATTATACAAAAAAATCAGTTTCGGACTGAATTGGAGAACATTGAAACATATATAAAAATGAAGATGCCAAATGTGATGTTCTGCACAGTAGTATCCATGTGTGCATACAATTTTATATACACTCAGTATGAATCAATTTACGCCTTCATTATTCACTCGATTGCTATTTGTTCATATATTTTTAACTTCGTATTTATGTGCCCCCAGATTGTCCGAAATTATTACACCAGAACGGTGGAGCGTGTTCCCCTATTTTTTCTCTTCTTCCTTTTTTTATATGCACTCATGGATGATCTGTTTGTGCTCGTGCTCCGCATACCCCTTGTGCACAAGTGGAACGCACTAGGTGATGATATCGTGTTTTTTATATTTTTCGTTCAATATTGTGTATATAAGAAGGGAAAGTCCCGGGTGGGCTCCGGCGAGGTGGCCGCGGCACCCCAGGGCGCCAAGCCGCAGCGCGAGTCTAAAAAGAAGAAGTGA

>Pv_6737|Plasmodium vivax

ATGATAAAATTCGCAGTGGGAATCATATGCTACTACATCGTGTACGGCTCATACCACGTTTACCAAAATGTAAGGACCCCCGTGTATGATCACTCAAGGGATGGGAAGGAAAGCGGAAAGGAGCAGGAGGGACCTCACGTGGGTGAGAAATATATCCATAGACCGTTCAGAAATTTTTTCAAACAAAAAGACAAAGTGGATTATCACCTGTACATGTCATGTGAAGAAGATATTGACCTGAACAGATACGTAAATGAGAAAGAAAAATATTTGAAGAAAAATAAAAATTTTATTAACGTGTATAATTTTAATAACGTTTCGTATGATTGGAGTTACAGTCATCCTGCAGGGGGGGGAGGAAACAAGTGGTGGAATGTCCTCTCGCCAAGGAAGTATCCTTCTGTTAAGCTGACCATCCCAAGGAAACTCATAAAGAAGAACAAAGAAATTTACCTGCACATAATTACCTACGTGAATGGAGAACTGTACAGGCACGGCTTCGTCACGAGCGCTCTCACGAGGGGGAAACGCGGGGGGAAGAAGTGGACGCGCAAGGAGAAGTACCTCTGGGAGCGCTTCCTAGACGAGGAGGAGGATGATGAAGAGGAGGAAGAAGAGGAGGAAGAGGAGGAGGAGGAAGATGACGAGGTAGACGAGGCGTATAACGGTCAATACGGCGGTCACTACACCGGTGAGTATAATGAGCAACACGCCGAACAGCACGCGGACGACGCGGAGGGACACAACGAAGCGGAAGAGGGGGACGACGACGACGATGATGAGGAGGAGGAGGAGAAGGAGGCCGAGGGGGAAAATGACGAGGAGACCCAAACAGGTGACGCTTCAAACGAGGGACAAAAAAAAAGCAGCGGCGTAGCAACCCATGAAGGCGTAAAGAAAAAAAATAAAAAAATGAAGGCAAAAAAAAGGCACGCGAAAAAAACGAAACAAAAAATTTTTTACATTCCAAAGAAGGTGAAGTTTGGACCAGTCATCGAACATAACGATATCAACGTAAATAAAATCGGCTTCTTTTCAAACATATTTTTGGATAAAGAAAATAGTGTGTACCTGTTGCCAACGTACTACAATGATCACCTGACTGCGGAAGATGAATATGAATTGCTTCAAATCGGAGGGGAGGGAGAGAAGGAAGACGTGGGGAAAAAAAAAAAAAAAAAAAACGCACAAGAAGATAATGAAATGTATTCGAAGGATAACCATACGCAGATGGGAAATGTAATTGAAATTGAATATTCGCCAATCAGCCTCCCCCAGTTCAATCTGTATAACATCATCATCTTTAATGTAAATTACGCGAAAGAAAAATATAAAATTGCCGCGTACGATTTGGATAGCTTGGTCATACACTTTTGTGGGAATATTTCTCTGTGCCTCATCATATGTCTCCTTTGCCTCATCCTTTTGCTAATCGATCTGGTGGCTCTCGTTTTTGATTTGACCAGCTGGAATCGGCTGAATGGGCTGTATTCCTTCTCGTCGGATGCCCTCCATTTCAAGTTCCTCTTCAGCATGTTCATATTTCTTTATCTGAAGAATAAAAGCAACTGCAAAATTTTGATGGTCTTTTGCGTGGCGAAGATGGCCGTCTGCCTTTGGAAGCTGCTGGACCGTTACGACATCGAGTTTATGGAGGTCCACCCGTACGTGTGCATTACGAGCAGCACCGGGGGGGGCGCAGGGAGTGCAGGAAGTGCAAGAAGCGGTGTAGGCGGCGGGAGCGGCAATGACAACCCAGGCAACGGCGGCGGCAGTGGCAGCGGCAGTGGCGGCGGCGCGGGGACGCACAAAAGGCAGTCGCGGAGTGAGGTGGAGGACCTCGAACGATATATAAAAATGAAGATGCCGAATGTGATGATCTGCACAGTAGTATCCACGTGTGCATACAATTTTATGTACACTCAGTACGACTCAGTATACGCCTTCATCATTCACTCCGTTGCTGTTTGCTCCTACATCTTTAACTTCCTCTTCATGTGCCCACAGATTGTCCGAAATTATCACACCAAAACGGTGGAGCGCGTGCCCCTGTTTTTTTTCTTCTTCCTTTTTTTATATGCGATCATGGATGACCTGTTTGCGCTCGTGCTGCGCATGCCCCTCGTGCACAAGTGGAACGCGCTGGGCGACGACCTCGTGTTTTTCATTTTTTTGCTGCAGTACTGCGTGTATAAGAAGGGGGACTCGCGGGTGGCTCCCGGCGAGGCGCCCGCCGCGCCCCCGGGCACCAAGGCGCAGCGCGAGTGCAAGAAGAGGAAGTGA

**1) Genomic sequences *P. falciparum* serpentine receptor PfSR10 (PFL0765w) orthologs:**

>PfSR10 (PFL0765w)

ATGGTGATATGGAAGGGCAATGTAAAAAATAAAATATTGTTTTTAATTTTTGTAGCTTATTTTTTTGTTTTTGTTAAGATAAGCAATGGTCAGTTGATTAAATTGGATGGTCAGAAAATTAACACGAATTATATTCTTTATGTTTTGAAAGGTTTATATATATTTGGAGAGAATGAAAGTCCCTATGTTTTATTAGGAAAAAAAAAGGATATGGATTTTAAGGCAGCTCATGCGATTTTTGAAAATGTGGGTATAAGTACGACAGATAATAAGAACACGAAATATTTTTCTTTTGAGATGGGCGACACGACAAGTGGTAATAATGAAAATAATAATAATAATAATAACGACGGTCATAATAATAATAATAATGACAGTCATAATAATAATAATAATGACGGTCATAATAATAATTATGACCATAATAATGACAGTACACTAGAGAATACGAACCTACCACAAAATAGTTACAACAATAATGGTAATAACGGAAATAATTCAAGTGAAAAACATAAGGATGAGGATGAAGATAAGGATAAATTCAAAATAAATTTATACAAAGATAATCCTTATCTACGTAAAAAAAAAGAATATAGATACTCTGAAGACGTAGATTCTTTTGTTACGTCTGAATTATTTTTAGAATTAATTATAATGAAAGAAAAAGATTTTAATAAACATTATTTACCAAAAGATCATGACATTTGTTGTTATATGCAAGAAGAAGGAATAGACGGATATGAAAAATATACATGTCCTGGCAAAGGGTACTTGAAAAGATATGTGGATGAAGAGCATATGTATTCGTTAAAATTACCTGTATATTTTATTAATGATAGGATAAAAGATGATACAAATAATAATAATAATAATAATAATAATAATAATAATAATAATAGTAGTAGTAGTAGTAGTAGTAGTTATTATAATAATATGTATAATTTGAATAATGGTAATGAGATTAATCATGAAAATTTAATAAATCATCTTAAAAATAAATTTGTTTATAATATTAAAGATACAGATGTTTATGCTTTATTTTTATCAAATTGTTTAGATAGTAAAAAATATGAACTACATTTACATGGAAATATCCATATTTTAAATGATTATGGTTATTTACCAGGAGATAAAATATCAAAATTAAATTTATATGTATTAAGTATGATAATATATTCTATTTATTTATTTATATGGTCCTATTTATTAATTAGAAACAAAAATTATGTTATAAAAATACAGATTTGGATTTTAGTATGTGTATTTTTATATTTAATAGAAAATATTTGCTTATTTTTATATTTCTTATCATATAATTTATATGCCAAAGTTAATAATGAATTATTATTTATATCTGTTTGTTCTAGTATCTTAAAAAATGTTTGTTCATATCTTCTTATATTATTAGGATCATTAGGATGGGGTATTGTTATTCCTACATTAGATAGAAAGACATTTATTAAAATCAAAATCCTTTTTTTCTTTTTTATTATTTTTGATTTTATTAAACAATTTGTAGATATGCATTTAACAGATACACAAATTAATACTGGATATTTTTTTTTCTGTATAATACCTGTTACAATTATCTATTCTATTATATATATATGGGTTTTTACTTCAGCTAGCCAAATTATTATACAATTAAATGAGGATAAACAATATGAAAAATTAAACATGTTTAAAAACCTTTTTAATGTATTAATATTTACTTTATTATTTTCGGTTATAGCATTTATAATAGACATAGTAGTAATGTTATATGTAGATAATAGTATATGGAATTTAAAAAATTATTTAAGTGAAGGAATTATTAGTTGTTTATTTTTAATTATATTAACAGCCATGTTTATTTTGTTTAAACCTTCTGATAGGCTTAAGAGAATATCTCATTTTACAGAAATTGGAGATATGGATGAAATGGAAGATTTTTCAAATTTCAAAAATTCTATTGAAGATATATCATAA

>Pk_979e07p1ca|Plasmodium knowlesi

ATGATATGGAAGTCTACTCAGAAAGTTAAATGCAAAACCGAGTTCCTGATCGTAGTGTTTCTTGTCCTGTTTGCAAGCATAACGAACAGCCAATTGATAAAATTGGATGGACAAAAAATAAGCACCAACTACATTCTATACGTTCTTAAGGGTCTATACATATTCGGGAAAAATGACACTCCATACGTCTTGTTGGGGGAGAAGAAGGACATGGCAACGAAGGGGCCGCATGCAATTTTTGAAAACATAGGAATCAGTACCACAGACAATAAAAACACGAAATATTTCAGCTTCGACATAGAGGAAAGCAGAACGAATGGCCAGGAGAATAACGAATCTGATGAGGAAAACAAGTCGGACGAGCACCCCACCTCGGAGATGAATCATAGCAGTGATGGGGATGACGACGACGATGATGAGAAGGATAAAAAGGATAAATTTAAAATCAACCTGTATAAGGATAATCCTTACTTAAGAAAAAAAAAGGAGTATATGCACAAGCACGAAGATGACGAAGTGCTAGACGCAGGTAACTTGTTTTTGGAAGTAATTATAATGAAGGAAAGCGATTTCAATAAGTTTTATTTACCAAAAGATACGAACGTATGTTGTCACATGGAAGAAAATGGTATGGATGGAAATGATTCATATACGTGTCCAGGAAGGGGGTACCTAAAAAGGTACGTAGAGGAGTCGAGTATGTATTCATTAAAATTACCAGTTTATTTTTTGAATGATAGAATATCGAACGACGATGAAATATCTCCTCTAGAGAATGAAGTAAATCATGAGAATTTTTTAAAAAAAATTCAAAATAAACATATATACAACATAGACGATACGGATGTGTACGCATTATTTTTATCAAATTGTTCAGATAGTAAAAAGTATGAATTAGAGCTACATGGTAATATACACATTTTGAATAAGTATGGCTATCTACCAGGGGATAAAATACCAAAGCTAAATTTGTACGTTGTGTGTATGCTTATTTATGCGATTTATTTGTTTACCTGGATATATTTACTAATAAGGAACAAACAGTTCGTTATCAAAATTCAGATATGGATACTGGTATGCACGTTTCTCTACCTGATGGAAAATGTCTTCCTCTTTCTGTACTTTTTGGTTTACAACTTACGCGCAAGAGTGAATAGCAATTTGCTCTTCCTTTCAGTATGTTCAAGTATATTAAAGAATGTATGTTCCTATCTTTTAATTTTACTGGGCTCTCTAGGCTGGGGATTAGTCATCCCAACATTGGATAAGAAAACGTTTATTAAGATAAAAGTGCTCTTTTTCTTTTTTATCATTTTTGACTTCATAAAACAATTTTTGGACATGCACTTAACCGATGCAGAAGTTAACGCAGTTTATTTCCTCTTCTGCATAATTCCAGTAACCATTATTTATTCTATAATTTACTTGTGGGTATTTACATCAGCTAGCAAAATTATCATTCAGTTAAATGAAGATAAACAGTACGAAAAGTTGAACATGTTTAAGAAGTTTTTTAACGTATTAATTTTTTCACTTATTTTTTCAGTCATTTCTTTTGTTATAGATATAGTCGTTATGCTTTTTGTGGATAATACCATATGGAGTTTGAAGTGCTACATTAGCGAGGGAATTATCAGTTGCTTGTTTTTGATCATCATCACAGCCATGTTCATGTTGTTTAGGCCCTCGGATAGACTCAAGAGAATTTCGCACTTTACGGAGATTGGCGACATGGATGAGATGGAGGACTTTTCACAATTCAAGGGCTCCATCGAAGACATTTCGTAG

>Pv_3938|Plasmodium vivax

ATGATGATATGGAAGTCCACTCAGAAAGTTAAATGCAAAAGCGAGTTCCTCATCGTAGTGTTTCTTCTCCTGTTTGCAAGCATTACGAGCAGCCAGTTGATAAAATTGGATGGACAGAAGATAAACACCAACTACATTCTGTACGTTCTTAAGGGCCTGTACATATTTGGGAAAAATGACAGTCCGTACGTTTTGCTGGGGGAGAAGAAGGACATGGCAACGAAGGGGCCGCACGCAATTTTTGAAAACATAGGAATTAGTACAACGGATAATAAGAACACGAAATATTTCAGCTTCGACATGGAGGAAAATAAATCGGACGAGGAACACAAGGCGGATGAGCACTCCACCTCGGAGAGCAACAACGGCAGTGATGATGAGGATGAAGACAGTGATGAGAAAGACAAAAAGGATAAATTCAAAATCAACCTGTATAAGGATAACCCGTATTTAAGAAAGAAAAAGGAATATATGCACAAGCATGAGGAGGACGAAGTGCTAAACACAGGTAACCTATTTTTAGAAGTAGTCATCATGAAGGAAAGCGATTTCAATAAGTTTTATTTACCAAAAGATTCAAACGTCTGTTGCCACACGGAGGAAAGGGGGATGGACGGAAATGATGCGTACACCTGTCCAGGAAGGGGCTACCTAAAAAGGTACGTCAAGGAGTCAAGTATGTATGCATTAAAATTGCCAGTGTATTTTTTGAATGACCGAATTTCGAACAATGATGGGATGTCTCCTCTAGAGAATGAAGTAAATCATGAGGAGTTTTTAAAAAAAATTCAAAGCAGACATGTGTATAATATAGACGAGACGGATGTGTACGCATTATTTTTATCAAACTGTTCTGATAGTAAAAAATATGAATTAGAGCTACATGGCAATATACACATTTTGAATAAGTATGGATATTTACCAGGAGATAAAATACCAAAACTGAATTTGTACGTTCTCTGTATGATCATTTATGCCATTTATTTATTCGCTTGGATTTATTTACTAATGAGGAACAAACAGTTTGTAATAAAAATTCAGATATGGATTCTGGTGTGTATATTTCTTTACTTGTTGGAAAATTTCTTCCTCTTTCTGTACTTCTTGGTATATAACATACGCGCAAGGGTGAATAGCAATTTGCTCTTCCTTTCAGTATGCACCAGCATTTTAAAGAATGTCTGTTCGTATCTTTTAATTTTACTGGGATCTCTAGGCTGGGGTTTGGTCATCCCCACATTGGATAAGAAAACGTTTATCAAGATAAAAGTGCTCTTCTTCTTTTTTATCATTTTTGATTTCATAAAACAGTTTCTGGACATGCACTTAACCGATGCGGAGGTGAACGCAGTTTATTTCCTCTTCTGCATCATCCCAGTGACGATTATCTACTCGATTATTTACTTGTGGGTATTTACCTCAGCTAGCAAAATTATCATTCAGCTAAATGAAGATAAACAGTATGAAAAGCTGAACATGTTTAAGAAGTTTTTTAACGTGTTAATTTTTTCGCTCATCTTTTCAGTCATCGCTTTTGTTATAGATATCGTCGTTATGCTCTTTGTGGATAATACCATATGGAGCTTGAAATGCTACCTTAGCGAGGGAATTATCAGCTGCTTGTTTTTGATCATCATCACCGCTATGTTTATGTTGTTCAGGCCCTCCGACAGACTGAAGAGAATTTCACACTTTACAGAGATCGGCGATATGGACGAGATGGAGGACTTTTCACACTTCAAGGCCTCCATGGAGGACATTTCGTAG

>Pb_5222|Plasmodium berghei

ATGGTGATATGGAAGGCCAATCCGAAAAATAAAAACTTATTATACCTTCTCTTCTTGTATATATTTTTTATAAGTTTTACAAATTGTCAATTAATAAAACTCGATGGGCAAAAAATCAATACAAATTATATACTTTATGTTTTGAAGGGTTTATACATATATGGAAAAAATGACGTACCATATATTTTGTTAGGAGAGAAAAAAGATATGAACAGTGCAGGACCCCATGCTATATTTGAAAATGTAGGTATAAGTACAACTGAAATAAAAAATACTAAGTATTTCACATTTGGCAGAAAAAATAATTCTGATCATAATGATAATGCCAACGAAAAAAATGACGAGGATGGGGAAATGGAGAAGACAAACAATAATAATTCCACTTCTTATGAAGAAGAAGATGACGAAAGTGAAGAAAAAAGAAAAAAAAAAGGATTTAAACTAAACTTATACAAAGATAATCCATATGTTCAAAGAAAAACAGAACACTCAGATTTGGAGGATTGGGATTCAAATGATAATATATCTGATTTATTTTTAGAAATAATAATTATGAAAGAAAGCGATTTTAATAAATTATATTTACCCAAAGATAGTAATATGTGCTGCTATACTGAAATGACTGGAATCGATAATAATGACAAATACACATGTCCAGGAAAAGGATATTTAAAGAGATATTTAGGTGAATCCGAATTTCATTCATTAAAAGTACCAATTTATTTTATAAATGATAGAATAGAAGATGATAATACTTCGTCAGGAAATGAAGTAAATTATAATAAATTTCTAGAACTAATAAAAAATGAGCATATATATAATATAGACAAAACAGATATATATACAGTATTTTTATCAAATTGTGGAGATAGTAAAGTTTATGAATTAGACCTACATGGGAATATACACATATTGAACAAATACGGATATTTACCAGGAGATAAAATACCAAAGCTAAATTTATATGTTTCTCTTATGATTATATATTTTATATATTCAATGATATGGTCCTATTCATTAATAAAAAATAAAACAAATGTTATAAAAATTCAAGTATGGATATCAGTGTGTATATTTTTATATTTATTAGAAAATATGTTTTTATATTTATATTTTATGACATATAATGTTCAGGCTAAAATAAATAATAATTACCTATTTATGGCAGTTTTTTTCAGTGTTTTAAAAAATGTTTGCTCTTATTTATTAATATTATTAGGGTCATTAGGTTGGGGACTAGTTATACCAACATTAGATAGAAAAACATTTATTAAAATAAAAGTGTTATTTATTTTTTTTATAATATTTGATTTTATAAAACAATTCTTAGATGCCCATTTAGCAGAAGAACATGTGAATGCTGTATATTTCTTATGTTGTATTTTGCCTATGTCTATAATATACGCCATTATATATATATGGATATTTATATCATCAAGCAAAATTATTATACAATTAAATGAAGATAAACAATATGAAAAATTGAACATGTTCAAAAACTTTTTTAATGTTTTAATACTTGCACTTATATTCTCAATTATTTCTTTAATTATTGATTTATTTGTAATGTTGTTTCCAAGCGATCAATTATGGAACCTAAAATGTTACATAAGTGAAGGAGTTAACAGCTTCTTATTTCTAACTGTCTTGTCAGCTATGTGTATGCTATTTAAACCCTCAGAGAGACTTAAGAGAATTTCTCATTTCACAGAAATTGGGGATATGGATGAAATGGATGATTTCTCTCATTTTAAAAATTCAATTGAAGATATTTCATAG

>Pc_3427|Plasmodium chabaudi

ATGGGGATATGGAAGGCCAATCCGAAAAATAAAAACTTATTATACCTTCTCTTCTTGTATATATTTTTTATAAGTTTTACAAATTGTCAATTAATAAAACTCGATGGGCAAAAAATCAATACAAATTATATACTTTATGTTTTGAAGGGTTTATATATATATGGAAAAAATGACGCGCCATATATTTTGTTAGGAGAGAAAAAAGATATGAACAATGCCGGACCCCATGCTATATTTGAAAATGTAGGTATAAGTACAAATGAAATAAAAAATACAAAATATTTTTCATTTGGCATGAAACATGATTCTCATCATGATGATAATATCAACGAAAAACATAACGAAGATGGGGAAGCAGAGAAGGCAAAAAATCATAATCACAATTCTTATGATGATGAAGATGATGAGAGTGACGAAAATAATAAAAAAAAAGGATTTAAACTAAATTTATATAAAGATAATCCATATGTTCAAAGAAAAACAGAACACTCAGAAACGAAGAGTTGGAATTCAAATGATAATACATCGGATTTATTTTTAGAAATAATAATTATGAAAGAAACTGATTTTAATAAATTATATCTACCCAAAGACACCAAGAGGTGTTGTTATACTAAGATGACTGGATTTGATAATAGTGATAAATACACATGTCCAGGAAAAGGATATTTAAAAAGATATTTAGATGAATCCGAAATGCATTCATTAAAAGTACCCATTTATTTTATAAATGATAGAATAGAAGATAATGATACTTCATCAGGAAATGAAGTAAACCATAATAAATTTCTAGAACTAATAAAAAATGAACATGTATTTAATATAGACAAAACAGATATATATACTGTATTTATATCAAATTGTGGAGATAGTAAAATTTATGAATTAGAATTACATGGAAATATACATATATTGAATAAATATGGTTATTTACCAGGAGATAAAATAACAAAGCTAAATTTATATGTTTCCCTTATGATTATATATTTGTTATATTCAATAATATGGTCCTATTCATTATTTAAAAATAAAACAAATGTTATAAAAATTCAAGTATGGATATCAGTATGTATGCTTTTATATTTAATAGAAAATATATTTTTATATTTATATTTTATGACATACAATGTTCAGGCGAAAATAAATAATAATTACCTATTTATGGCAGTTTTTTTCAGTGTCTTAAAAAATGTTTGCTCATATTTATTAATATTATTAGGGTCATTAGGTTGGGGGCTAGTTATACCAACATTAGATAAAAAAACATTTATTAAAATAAAAGTCTTATTTATTTTTTTTATCATATTTGATTTTATAAAACAGTTATTAGATGCCCATTTAGCAGAAGAACATGTAAATACGGTATATTTCTTATGCTGTATTTTGCCTATGTCTATAATATACTCCATTATATATATGTGGGTATTTATATCATCAAGCAAAATTATTATACAATTAAATGAAGATAAACAATATGAAAAATTAAATATGTTCAAAAACTTTTTTAATGTTTTAATACTTGCACTTATATTCTCAATTATTTCTTTAATTATTGATTTATTTGTAATGATGTTTCCAAACGAACAATTATGGAATTTAAAATGCTATATAAGTGAAGGTGTTAATAGTTGCTTATTTTTAACTGTCTTGACAGCCATGTGTGTTTTATTTAAACCCTCAGAGAGACTTAAGAGAATTTCTCATTTCACAGAAATCGGCGATATGGATGAAATGGATGATTTCTCTCATTTTAAAAATTCAATTGAAGATGTTCCATAA

>chrPyl_00625|MALPY00625|Plasmodium yoelii

ATGGTGATATGGAAGGCCAATCCGAAAAATAAAAACTTATTATACCTTCTCTTCTTGTATATATTTTTTATAAGTTTTACAAATTGTCAATTAATAAAACTCGATGGGCAAAAAATCAATACAAATTACATACTTTATGTTTTGAAGGGATTATATATATATGGAAAAGATGACGCGCCATATATTTTGTTAGGAGAGAAAAAAGATATGAACAATGCAATCCCCCATGCTATATTTGAAAATGTAGGTATAAGTACAAATGAAATAAAAAATACTAAGTATTTCACATTTGGCCGAAAAAATAATTCTGATCATGATGATAATGTCAACGAAAAAAATGACGAGGATGGGGAAATGGAGAAGGTAAACAATAATAATTCCACTTCTTATGAAGAAGATGACGAAAGTGAAGAAAAAAAAAAAAAAAAAGGATTTAAACTAAACTTATACAAAGATAATCCATATGTTCAAAGAAAAACAGAACACTCAGATTTGGAGGATTTGGATTCAAATGATAATACATCTGATTTATTTTTAGAAATAATAATTATGAAAGAAAGTGATTTTAATAAATTATATTTACCCAAAGATAGTAATATGTGCTGCTACACTGAAATGACTGGGGTCGATAATAATGACAAATACACATGTCCAGGAAAAGGATATTTAAAGAGATATTTAGATGAATCCGAAATGCATTCATTAAAAGTACCAATTTATTTTATAAATGATAGAATAGAAGATGATAATACTTCTTCAGGAAATGAAGTAAACCATAATAAATTTCTAGAACTAATAAAAAATGAGCATATATATAATATAGACAAAACAGATATATATACAATATTTTTATCAAATTGTGGAGATAGTAAAATTTATGAATTAGATTTACATGGGAATATACACATATTGAATAAATACGGATATTTACCAGGAGATAAAATAACAAAGCTAAATTTATATGTTTCTCTTATGCTTATATATTTTATATATTCAATAATATGGTCCTATTCATTAATAAAAAATAAAGCAAATGTTATAAAAATTCAAGTATGGATATCAGTGTGTATATTTTTATATTTATTAGAAAATTTATTTTTATACTTATATTTTATGACATATAATGTTCAGGCTAAAATAAATAATAATTACCTATTTATGGCAGTTTTTTTCAGTGTTTTAAAAAATGTTTGCTCTTATTTATTAATATTATTAGGGTCATTAGGGTGGGGACTAGTTATACCAACATTAGATAGAAAAACATTTATTAAAATAAAAGTGTTATTTATTTTTTTTATAATATTTGATTTTATAAAACAATTCTTAGATGCCCATTTAGCAGATGAACATGTGAATACTGTATATTTCTTATGCTGTATTTTGCCTATGTCTATAATATACGCCATTATATATGTATGGATATTTATATCATCAAGCAAAATTATTATACAATTAAATGAAGATAAACAATATGAAAAATTGAACATGTTCAAAAACTTTTTTAATGTTTTAATACTTGCACTTATATTCTCAATTATTTCTTTAATTATTGATTTATTTGTAATGTTGTTTCCAAGCGATCAATTATGGAACCTAAAATGTTACATAAGTGAAGGAGTTAACAGCTTCTTATTTCTAACTGTTTTGACAGCTATGTGTATGTTATTTAAACCATCAGAGAGACTTAAGAGAATTTCTCATTTCACAGAAATTGGAGATATGGATGAAATGGATGATTTCTCTCATTTTAAAAATTCAATTGAAGATATTTCATAG

**1) Genomic sequences *P. falciparum* serpentine receptor PfSR12 (PFD1075w) orthologs:**

>PfSR12 (PFD1075w)

ATGATAAGAAGAAAGTGGAGTAAAATAAAATTGGCAATATATTTTATTGCATTTTATTACTTAACAAAAATAGATGAAAAATGTTTATTAATAAAAGAGGGAGAATTAAATTTGTCTATTACCAATGTACATGATAATAATTATATGATATCTGAAAAATATAATAAGTATTATATATTATCATTATTATATAAATTTGGAGGAATATTAAAAGAAATATATGATAAAAAAATATTTGTTAAAAATAAGAGTTGTAATAATTTTATGGTATCTTCAAAAGTTATATATGGTTTATATAATGATATGAATTATAGCAAATATTCAAATTTTTGTTTTTCCAAAAATGCAAATAATGGTGTTGTTATTTTATCTAATTTGTATGTCCCTAATACAAAATTTTTAATATTAGATAAAAGTGATGATGAAATATATAATTATGGAAAAAACAAAAATGGAAAAACATGTGAAGATTTAGAAAAAATTGCATTATTTGTACACCCCTTAAATGATATTCCTCCTGAGCTTATGAACAAAACATATTTTGTATACCAAAAAGATATCGAGAAATCGCTCACAGATAAAAAATTAAATTTTATTTTATTAAATTGTGGGAATAAAATTAAAAACGCATTCAAAATTGAATTTAAAAATAATATGAACTTTTTGAAAAATCATTTCTCATGTGAAGAGCAAGGTATAACATGTAAATAAATACAAATATTATATTGAAATGTTTATACGATTTTTAATTTATACATTGCATATATATATATATATATATATATATATATATATATATATATATATATTTATATTTATATTTATATATATTTATTTATTTATTTATATATTTATATATTTATTCATTTATACCTATTTGTACACATTTTAGGCCTTTTTGAAATTCACATGCTGCTGATAGTATTACTATTTGTCTTGTCCTTGGTATATTATAGAAAAAGAAAAAACTTAAATAACACAAATAATGTATTGAAGGAAGCTATACATTGTTCTTATTTATTCTTCCTGCTTTCTAATATATTATATTTTATACATTTAATATCTTATGCATTCAATGGATCTGGGTTTAGTATTCTCAAGGTACTAAGCCAAATATACGAAGCCATTTTTGATTGTTTCATTCTTGTAATAATATATTATATATTTAATAATGACATGCAAAAGAAAAAAGAAGAAACAATAAGGGTAGCTTTTACATATTCCATATTAAAATTTATTTACATATTATTTGAGATACAAAATAATCAGGAATTGGATTTATATTCAACTTTACACTCAAGTGAGCAAACACGAAAATATTAAATCTACATAACAAACGATTATATATATAAATATAAATATATATATATATATTTATTTATTTATTTATGTTTATTTTCCCGTATAGTTGTGGCTCTACCCTTTGTTGTTTATCGTATAATAGTTGCAGGTAAAAATTAATGAAAAGAAGATTCTTAAATAAATAAATAAATAAATAAATAAATATATATATATATATATATATATATATATATATATATATGTACGTATGTATGTTCCTCTTTTAATCTCTTCAGTGTTAAATTATGATAACAGCAAAAAATTGCTCAAGGAAAAAACACAAGTGGATGAAAAGTTCTATGTCCTTTTTGACACATTTTTGTTAGTTAAAAAAAAAAAAAAAAGGAATATATATAAATATATTTATTTATAAATTAATTATATTCATCTTTATTTATTTTGAGATATATATTGTATATATAACATATATATATATATATATATATATGTATATATTTTTTTTTTTTTTTTTTTTTTTTTACAGTTATAATTTATGGATATTGTCAATACCTGTTCAATATTTCTTAATGAAAAGCTTTTCATTGTAAATTAGAACATATGCATAAAATATATATATCAAATTTTTCATTGTATAATTTATTACAATACTATTATTCTTATTTTATTTTATTTTATTGTTATTTCTTTTTTTCTTTTTTTGTTAGGCACTTCACCCATTTGTTCGTACACTTCTTTAACCTTTACATATTGATTTGTAATAAATAAAATAAATACAAAAAATATATGACAAAAAAATAGAAAAAAAAAAAAAAAAAAAAAATTGTTATTATGAATATGTAAAATTATAAATATATGTACACATAATATATATAAATATAAATATAAATATATATATATATATATATATATATATATATATATATATTTAATTTGTTATTTTTTTTTTATTTGCTTAAGATTTGGTTTACAACTTGTCAGAGGAGAAATTTGAAGTTTTGGAATCGAAACATCCATATTTAGATTTAAATTAA

>Pk_961e09q1c|Plasmodium knowlesi

ATGGCGATGCCAAAGGGGGGATTTTTAACTGTTCTGTTTTTCCTACTATGTATTGCCCACTGGGATGGGGGATTTCCTCCCCCGGACAGGGACTGCCGACAGAGTTGTCCAAAGTTGTGTCCAACCGATGGGGAAAGCTACACGTGGGGTATATGGCCACGTAGTTCTGTCCCCATGATGAATGTTAAAGGAAGAAGTATTGCGAGTGGCTTTTACGCAACAGGGAAGGTCATATATGGGTTGAAGGAGGAAAGAAACTTTAGTGTGTATGGAGATTTCTGTTACAGTAGAAGTCGACATACAATGGGAAGAAATGGAGTGCTTATTGTGACAAGTTCGTATATCCCCAATACTAAATTGTTAATCCTGAAGAAGGTGGAGGAAGACATTAACAACTACATGAATGGGAAGAACGGAAACAGATGTGCAGACTTAGAGAGGAAAGCCTTGTTTGTGCACCCCTTTGATAATACCCCCGAGGGGAATTTACCGAATTCGTATTCCCTTTATGAAGAGGATATCGGTGACAATTTGAAGGATACCCCATTGAATTTTGTCCTACTCGCTTGTGGACGCCACGTGAAGAATGCCTACAAAATTGAGTTCCGAAATAATGCGCACTTTTTGAGGAATCACTTTTCTTGCGAGGATCAGGGTGCGCAATATTGAAAAAGGAGTTGAGAAGGCGTATTTCCGTTTTTCCCAATTCTGGCTCTCCACTTGTGTATGTCAGTCCAACAGTAGCACAACTTTACCGTTCCTCACCCCCACTCGTAGGGCTCATCGAGATCCACTTTCTCCTGGTGGTCATCTTAGTTGTCCTCTCCCTTGCGTACAAATCGAGGCAAGATTCATTGAGGGGAGCGCACAGTGCCATGAAGGAAGGTATCCACATGTCAGTGATGTTTTTTGTCCTCTCTAACCTATGCTACCTCATACATATATTCTTTTACGCATTCGACGGGACAGGGTTAACCTCATTAAAGGTGCTCAGTCAGATGGGTGAATCCATATACGACTGCTTCGTCATGACCATCATTTTTTACATTATGTGTTGCACAATGGATAGAGAGAAACGAAGGAAAGATACCTTCCGAACTGCGCTCAATTATGGTGTCCTAAAATTTTTATACCTTCTGGTGGAAATGCAAAACCAGGAAGACTTGAATCTCTACGCATCTTTGCATTCAGGTTGGGGGGGGTTAGCAAACTATGGTACCGTTACTTCGCGAGTGGAGGAAATGCCCACGCCTTCTTATTCTCTTCCCCTTTCACGTAGATATATTCATCCACACTCACGCATGCACATATATATATATATATATATATATATATCTACCTTTTTTCAGTTGTAGCTCTACCTTTTGTGCTTTACCGCGTGATCATAGCAGGTTTAGATTTGCTTCTCCACCCTAGAGTCTCACGCTATCTGGGGAGGGACAGTACATCCCGGGTGATGTCCCTTACCATGACGTGATAAATTATAACCTGTTCTCTTTCGTTCTTATCACGTTAGCAACGATTTACAGAAACTACAAGCGACTGCTGATGGAGAAGACTTCAAGGGAGGAAACCTTCTTCATCTCGCTTCACATGTTTTTGTTAGTTAATGAGGAGGGGTAACTCACAAACGGGAGTTATCTACGGGGGGGGGCGTGTGCCAGTGTTTTCCAGGCTTTACAGACGCGCCCTTTCTCTTATGTAGAAACTGTCCCCTTTTTTTCCATCGGGTCCTCCATCTCTGCAGATACAACCTCTGGATTTTGTCCATCCCGGCATATTACCTGCTGATGAGCAGAGCTTCGATGTAAAAAAAAATGGGAGATACGCTACTCTTGTTGTGGGGTGTGCATAGAGGACATGAGTGGGATGTAGTGTGCCACCTCATTGTCCTAGCCAGAGACGAATACGTACGTGCAGGCATCCCCCCCACAGGGGAGAAGCTTCTCCCATTTCCCCCTTCCAAGGATACGCACAAAAATGTTCGTTTTTTTTTTTTTTTTTTTCTTCCATCCGAAGCCATTTCACCCACCTGTACGTGCACTTTTTGAATCTTCTTATTTTGATATGTAATAGGGGGAGCAACCGAGCTGTGCATAGACCCGTTGCACTGACGCGTGGATTAGTCGGGCGTTTGCATTTTTATGTGATGCTATCTACGCAACTTCACTATGCTATATAGCTAGCTTTATTACTATTATTATTATTACTATTACTATTATTATTATTATTACTATTATTATTTGTTTTTTTTCCCTTCATTTGTGTATTTTCACCTGAACAGTTCATGCAAAAGTTAGCTATTTTTTTTTTTTTTTTTTTTTTTTTCGCTTTTTTCAAGATTTGGTGCATATCATTTCGGAGAAGAAGTACGAAGTCATGGAATCCAGGCATCCCTACTTGGATATGGAGTGA

>Pv_6872|Plasmodium vivax

ATGGCGATGCCAAAGGGGGCATTCCTAACGCTTGTGCTTCTCCTCCTGTGGGCTTCGCCCTGGGATGGGGGGTTCCCACCCCTGGAGAGGAGCTCCCCACATGGTTGCCCGCAGGTGCATCCAAGCGAGGGGGGAAGCTGCACATGGGGGATGTGGCCACACAGGTTTATCCCCCCCTCGCGTATTAAAGGAAGAACGAAGGCAAGCCGCCTCTACGCAGAGGGGAAGGTCATATACGGGTTGAAGGAGGACAAGGACTTCAGTGTGTACGCTCACTTCTGCTACAGTAGGGGTCGGTGGGAAAAGGGGAAGAAGGGGGTGTTCATTGTTACGAACTTTTATATCCCCAATGCGAAGCTGTTAATTTTGGAGCAGACGGAGCAGGAGATGCACAGCTATGTGGATGGGAAGAACGGAAACACGTGTGCCGATTTGGAGAAGAAGGCTTTGTTTCTGCACCCCTTTGATGACATCCCCCCGGGGGATTTAAAAGACTCCTATTTTGTTTATGAGGGGGACGTTGGTGACCATCTGAAGGGCACCCAGTTGAGCTTCGTCCTGCTGGCCTGTGGGCGCCAAGTGAAGAACGCCTATAAAATTGAATTCAGGAACAACACGCACTTTTTGAGGAACCACTTTTCTTGCGAGGAGCAGGGTGCGCAATCAGGCGGCGGCGAAGCGGTGCTGAAGCGGCGTTACTGCCGCGCCCATTCTGCCTTGTCACCTCTCCGCCTCACCACCGCTCCCCCCCTCCACATCTCCACCTCCCCCCCGCAGGGCTGATCGAGATCCACCTCCTCCTGGTGGTCATCCTAGGCGTCCTCTCCCTCGCGTACAGAGCGAAGCAGGAGTCGTTAAGCCGAGCGCACGGCGCCCTGAAGGAGGCCCTCCACATGGCAGTGATCTTTTTTGCCCTCTCCAATATATGCTACCTTATACATATCTTTTCTTTCGCTTTCAACGGAATAGGGTTCACCTCACTGAAGGTGTTCAGCCAGATAAATGAATCCATTTACGACTGCCTCATGCTGACCATCATTTTTTACATTATGGGTTGTACCATCGATAAGGAGAGACGGAGGGAAGACACCTTCCGAACGTCGCTCACCTATGGCGTCCTCAAATTTTTGTACCTTCTGGTCGAAATGCAAAACCAGCAGGAGTTGAATCTCTACGCATCGTTGCACTCAGGTTGGGGAGCGGCAAACAATCGGATGGTTCCTTCCGAAGTGAAGAGAGAATCCACGCCTTTGCGCTCTTCTTCCCATTTCACGTCGATTTGTTTTTTCTCCCCGTTTGCAGTTGTGGCTCTACCGTTTGTGGCTTACCGGGTGGTCACAGCAGGTTGGCTTGGCTTCTCCGGCGCACCTGGGAGGGGTGCTTCACAGGTGATACCGCCTCACCTGGCCTCCCCTGGCCTGCCCATTTGCATCCCTTCCCCTTCGTTCCACCCACCGCAGCAATGATTTACCGAAACTGCAAGCGACTGCTGATGGAGAAGACCCCGGGGGAGGAGACCTTCTTCATCCTGCTCCAAATGTTTCTGTTAGTTAATTGGGGGTGTAACTCAGGGAAGTTACCTCCTCGGGTGCGTCTCCCAGTGTGTGGCATGTATAATCCACCGTCATATTGTTTCCCAACCTTTGCAGATGCCCCCATTAGAAATTGCCTCCTTTTTCCACGGGGCCCCTCAACACTGCAGATACAACCTCTGGATTTTGGCCATCCCCGCGTACTACCTGTTGATGAGCCAAGCTTCGGTGTAAAAAAAAGGGAGAGACGCTGCTCCACTCTTGTTGTGGGGTGTAGAGGCATGAGTGGGATGTGTTGAGCCCCCCACTGTCCCATCGAGAGAGTGCATAAAACCTACGTATGGGCATCCCCCCAAAGGGAGAAGAAGCTGCTCCGGTGTGACCCTCCAGGGGTACGCAGCGAACTGTTCACTTTTTTTTCCTTTTTTTCCCGAAGCCATTTTACCCACCTGTACGTGCACTTCTCGAACCTTTTAATTTTGATATGTAATGGGAGGAGCGGTGGAGCGGTTCAGCGGTTGAGCCGTTCAGCGGCGGAGCTATGCAGACCCGTTGCAGTTACTCGTGCATAAGTCCGCGCTTGCATGCCCATATGATATGCCATCTACGCAACTGCGCTCCGTCATCCGCGTGTTTTTTTTTTTTTTTTTCTTCTCTTCTCTTTTCAAGATTTGGTGCACGTCGTTTCGGAGAAGAAGTACGAAGCCATGGAATCCAGGCACCCCTACTTGGATATGAAGTGA

>Pc_6587|Plasmodium chabaudi

ATGAACAAGCTATGTCAAAGGAAAAAGCTGCTTGTCTTTTTGTTTTTTTTATTGTACATATCAAATTTGGGTAATCAAAATTTATTACAATACGAAGATTCACATATAATTGAGCACAATGAGAGGGGCAAATATAGCTACTCGATATTTTCTCCGCTCGTTGAATATATTAAGGGGAGTAATAAAATAGAGGAAAGAAAATCATTTTCTTTTTTTGTATCATCAAAAGTAATATATGGATTATATCATAATAAGAATTATAGTAAATTTTCTGACTTTTGTTTTATTAAAAAGAATAATGAAAAAAAAGGATCTGTTATGCTTTCAAATTCGTATTCCCCTACTACTAAGCTTTTAGTTTTAGATAAAACAGATAATGAAATTTATAATTATACACAAAATAAGAATGGAAAGAAATGTGAAGATTTAGAAAAAGAAGCATTGTATGTATATCGATTTAGTGATACACCACAAGAAAATATAAATAATAATTATGTTTTTTATAATAAAGATATAGATCATCAGTTAGAAAATAAATCGTTAAATTTTATAATACTTCATTGTGATACTAAATTTAAGAATGCTTTTAAAATCGAATTTGTAAATAATGACAATTTTTTGAGAAACCATTTTTCATGTGAAGAACAAGGTATGTAGACAAAACTAAAAAATGTAACTATTTAAAATTTTTGCTTTTCATACATGTCACAATGTTTTATGGGGAGTGGAAATTACGAGCGGTGTTATATTAATAAAATTGCATATAAATAGGTGTGCCGATTTTTCGATTATTTTGTTTAATCCTTTTTAACATTGCTTAATTCCCTTCAGGCCTCATCGAGATATACATGCTGTTGTTTGTAATATCGACCGTTTTGTCGTTGGTATATGTTCGAAAGAGAAGTATGTTGAATAATGCGAATGGGGCATTAAAAGAATCGGTTCATTTTGGAGTTTTGTTTTTTTATTTTTCAAATATTTTCTATTTAATTCATATATATTCATACGCATTTAATGGGACAGGGTTTAGTATACTAAAAGTTTTAAGCCAAATATATGAATCAATTTTTGATTGTATAACATTAACAATAATTTTTTATATAGTAAATACTATTAATAATAAAAAGAGAAGGAAAGAGGATACTATAAAAACAGGATTTATATATTCCATACTAAAATTTTTTTATATTCTATTTGAGATGCAGAATCACCAAAGTCTGAATGTATATTCAAGTTTGCATTCCGGTTCGAATAAAAATTGAAACTACATTTATCTATATCTATTTAGAGCATTTGAAAGCTCAAATTCGTAAATATACATAAAAAAAGCTACGATTTCACTTGCTTACTTATTTATTATTGGTTAACCTTTTTAGTTGTAGCGCTTCCTTTTGTTAGCCACAGAGTGATTATTTCTGGTAAGGACCATAAACATGATTTTGACATATTATAACGTGAATATTTATTATAGACCTATATAAGTATGTCTATCTATTTTTTCTCAACAGTGTTAATATATAACAATTGTAAAAAATTGTTGAAGGAGAAAACAAGTGCAAGTGATAAAACCAGGCTCCTTTTGGATGCGTCTATGTATGAACCGAAAAAAAAAAAAGCCATGTCACAATGGGGAGTCGCAACATATGCTATAACCATTTTGACATACTAACCAATTTTATTATGCTTTATAAAAATAACTTTACTTTAACAATTATTATGTTTGCATATTTTGTTGCAGGTATATCGCATGGATTCTCTCTATTCCGTTTATTTATTTTTTTTTGTGGAATGCTTCAATGTAATAATACGAAAGCTAAACGAGTCGACAAATAATGGCATATTTTGCAAAGTCCACCCATGATTAATTGTATTCATAATAATTTTCTTTTCTTCTTTCTTTCACGCATTTAGGCACTTTACCCATTTGTTCATTCATTTCTCCAACCTATGCATATTAATATGTAAGAGTGGAAAAAATCGAATACAAATTTTTACACCATTTGGTTTAATCATGCTCTATGATTTGTTATGCGATTTGCTCTACGATTTGTTATGCGATTTACTCTACGATTTTATTATATGATTTGCTAATGATTTTGCTTTTTTGAAGGTTTGGTATACAACATTTCAGAGAAGAAGTATAACAGTTTGGAGTCCAATCACCCATACATAGATATGGAATAA

>chrPyl_02193|MALPY02193|Plasmodium yoelii

ATGAGCAAGTTTTATCAAAGAAAAAAGGTATTTGTCTTTTTGTTTCTTTTATTGTACATATCAAATTGGTGTAATCATATTTTATTACAGTACAAAGATTCATATATAATTGAGTATAATGAAAAGGGTAAATATATGTATTTAATTTTATTTTGGCTAGTTGAATATATTAATGGAAGTAATAAAATTTTGGAAAGAAAACCATTTTCTTTTTTTGTATCATCAAAAGTAGTATATGGATTATATAATAATAAAAATTATAGTAAATTTTCTGATTTTTGTTTTATTAAAAAAAATAATGAAAAAAAAGGATCCGTTATTCTTTCAAGTGCATATTCCCCTACTACTAAGCTTTTGATTTTAGATAAAACAGATGATGAAATTTATAATTATACAGAAAATAAGAATGGAAAAAAATGTGAAGATTTAGAAAAAGAAGCATTGTTTGTGTATCGGTTTAGTGACATGCCACAAGAGTATATAAATAAAAATTATATTTTTTATAATAAAGATATAGACAATCAATTAGAAAACAAGTTGCTAAATTTTATTATACTTCATTGTGACACCAAATTTAAGAATGCATTTAAAATCGAATTTGTAAATAATGACAATTTTTTAAGAAACCATTTTTCATGTGAAGAGCAAGGTATGAAGAAAGTTATTTCGCTGTTGTTTGTCCGCCTTTTTCATTTCGCCGTTGTCATTTCGCCGTTGTTTATTTTTTTATTTTTTTTTTTTTTTTTTTTTCCTCTTATTTTTATGTAGGGCTGATCGAAATATCCATGCTACTGTTCGTAATATTATCAGTTTTGTCGCTGATATATTTTCGAAAAAGAAATATGTTGAGTAAAGAAAATGGAACATTAAAAGAGTCGGTTCATTCTGGGGCTTTGTTTTTTTATTTTTCAAATATTTTTTATTTAATTCATATATATGCGTATGCACTTAATGGGGCAGGGTTTAGTGTACTAAAAGTTTTAAGTCAAATATATGAATCAATTTTTGATTGTATTATTTTGACAATACTTTTTTATTTAATAAATAGCATTCATAATAAAAAGAAAAGAAAAGAAGATACTATAAAAACTGGATTTATATATTCCATGTTAAAATTTTTTTATATTTTATTTGAGATACAGAATCATCAAACTTTGAATGCGTATTCAAGTTTACATTCCGGTTCGGGCAAAAATGAAAATGAAAATGAAAATAAAAATAAAACATATATATTTGTGTTGGGANGTGTTTACATTTATTTACTCATTTATTATTGATTAACCTTTTTAGTTGTAGCATTCCCTTTTGTTAGTCACAGAGTTGTTATTTYTGGTAAGAATCATAAATATATTTATTTTTATTAAAAAAATCACCATTTTGACATATTATAATGTGAATATTTATTTATTATAGATCTATATAAATGTGTCTATATAWATTCTTTCCTTTTTAGTGTTAATATATAACAATTGTAGAAAATTATTGAAGGAAAAGACAAACATAGCTGATAAAACTAATGCTCTTTTGGATGCATCTATGTATGAAAAAAAAAAAAAAAAAAAAAAAAAAAATAGTATCACAATGGAGAGTCGTAATATACATACTATAACCATTTTGAGATATTGAATTTTATTCTATTTTACAAAAATAAATATTTTTTAACATTTCTTATGTATACATATTTCCTTATAGATATATCGCATGGATTCTGTCCATTCCGTTTATTTATTTTTTTTTGTGGAACGCTTCAGTGTAATAATAGGAAAGATAAAATAAGTTGATAAATAATGAAATTTATCCCCAAAACAATATATAGCTATTTGCAGAGTCTATTGCGTGCTTTGTTCATAAAACGGTTCCTTCCTTTTTTCATTTCTTTCCTTTTTTCACACATTTAGGCACTTTACCCATTTGTTCATTCATTTTTCCAACCTATCCATTTTAATATGTAATAATAAAAAAAATAATATTTTTAATTTAGATACGATTTTATTTAACCAGTTTATATGAAATTATTTTATTTGGTTTTATTTGGTTTTATTTTTGAAGGTTTGGTGTACAAAATTTCAGAGAAAAAGTATAACAGTTTGGAATCTAATCACCCATATATAGATATGGAATAA

**1) Genomic sequences *P. falciparum* serpentine receptor PfSR25 (MAL7P1.64) orthologs:**

>PfSR25 (MAL7P1.64) ATGGCTAAAAGGCACAAATTAAAGATTACAATTTTGTCAATATTTTTTTTTGTGATATTTACGGGGATTCATACCGTTTTCACAGCATTCAATAGAAAAGATTGTAAGCTTAAATATATCTAAATATTTGTATATATATATGTATTAAGATGAAAAATATCGAAAATAAGATATATAATATAATTTCTATATTCAATACATTATTATGTATTATATAAGTTTTAGTCCCCTTGACATTTTTCTGCATCCCTCATATTATTTTGAACTCATATTATATTATATATATATATATATATTTTTTTTATTTTTCAGGGTTAAAGTTTTATACTAGTTGCTTTGGTACGGGAGAAGTAAAATGGGAACTGCTAGCCTTGTTAACAGTTGTAAATATGCTTCTTTTATTATTAAACGTGAATTACAAAGAGAATATAAACCATTTAAATAATAAAAAGAGTGAGACAAGTGATATAAATGATGATTTAATAAATGTTGATATGCATGAATTTAGTAATAATGAAAAAGATGAAAGTAGTGATGAGAATGAGAAAGAGAAGAAATATAATAAATTAAAAATAAGATATTTATATAATGTTAGTAATTCTATTATTTGTTATTATAGTTTATGGATCTTATGTTATTATTTAATATACTTTTTATGTTTTTTAAGTTTTTTATATGGTATAAGAAAATTCAATAATAATGTAATAAATATATATACCTTGAGGACATGTAAAATTGATAAATTAACAAATTATATATTATCAGAAAATACATTTATTAGTTTATATTGGGCTATTATAAATTTTAATGTTTTTATGAGTAAATATACAGATTCCTTTTATGTAGTTAATTATTTTAAATTAAATTTTGAATTTAGTAATAGGAAGAAAAAAACACTTTTCATTCTTAATTATATGTATCAATTATTATTATTATCCTACACTATATACAAAAATATTACTTTATATACTAAAGGAGAATATAATTTAAATCAAATCATTTGTGCTCTTATATTTTTATGCCTAATATTATATACCATTTTAGAAATTACTTATGTGTTAGAAATTAATAGACCATGTTATAATGTACAGACCAAATTACCTTTCCATTATGTGTGGGCCATAATTTATTTATTTATAATTTTCACATCATCAGTTATTTTTTATTTTTCCGTTTTTAGTTATTCTATAAAAGACCAATTTGTGAATTTCCAAATTACCTTGTGGCTTTTTTTTATCTCGCTAACATATATAAAAAAAAATCAGTTATTCATCAAAATATGA

>Pk_347d07p1c|Plasmodium knowlesi

ATGGCTAAGAGGCATAAATTGAAGATTACCATTTTGCCTATATTTTTTTTTGTAATATTCACCGGGATTCACACCGTTTTTACCGCCTTTGACAGGAATGACTGTAAGTTAAATGAAATGAAAAAGGGTCCTCGTGTGTACGGCATCATGGGGTATTATGCCACCACGCGTGTCTGTGCATTATGCATGGCCTAGTTTTTACCATGTCATTCTTGTATACCACATTCGGCTTGTGCAAATGAGTCCTCCATGTTCAGTGGATGTCTAGTTTTATGTTTAAGAAGAATGGCGTTCCGCTAAGGTCCACTGTACCTTTTTCATTTATGGGTGTGCACTTATCTATTCGCACAATGGAATGCTCTCTTTCCGCGCTATGCAATCGTTGCACCTGTGGATACAGATTCATCTCCATCGTGTGTTACCCCCTCTTTTTTTTTTCCCCCCCTTAGGGTTAATGTTTTACACGAGCTGCTCTGGCTCAGGGCAGGTAAAATGGGAACTGCTGGGGGTCTTGACCATTTTGAACAGTCTGATTTTATTGCTAAATGTGAACTACAAGGAAAACATAAACCATTTAAATAACAAGAAGAGCGAAACGAGTGACATAAATGACGATTTGATAAATGTTGACATGAACGAGTTTAGCAACAATGAGAAGGACGAAAGCAGCGATGAAAGTGAAAAAGAGAAAAAGTACAACAAGATAAAAATAAGATACCTATACAGCATTAGCAACTCTAGAGTCTGTTATTATAGCATGTGGATCTTATGTTACTATCTGATATATTTTTTGTGTTTCCTGAGTTTTTTATATGGTATTAGACTATTCCAGAACAATTTAATTAATATATATACAATCAGGACATGCAGAATTGATAATTTAGCAAATTATATTTTATCTGAAAATACCTTCATAAGCCTATACTGGGCCATTATCAATTTTAACGTTTTTATGAGTAAGTACACAGACTCCTTCTACATTTCAAATTATTTCAAATTGAATATACAATTTAGCACAGGGAAGAAGAAGCTTCTTTTTTTCTTAAATTATGCTTATCAAATATTACTAGTAAGTTATACGATTTATAAAAATGTGGATCTGTATAATAAAGGGCTGTACAATTTGAATCAAATTGTGTGTGCCCTCATCTTCCTCTGTCTAATCCTCTATACTATTTTAGAAATCACATATGTTTTGGAAATAAATAAGCCCTGTTATTATGGTGTTACCAAGTTGTCTTTCAACTACATTTGGGCCATCATATACCTATTTGTGATTTTCATTTCCTCCGTCATTTTTTACTTTTCTGTTTTTCCTTATTCCATCAAAGACCAGTATGTGAATTTTCAGATCATGCTCTGGTTTTTTTTTATTTCCCTAACTTACATAAAAAGGAACCAATTGTTTATAAAGGTTTGA

>Pv_6727|Plasmodium vivax

ATGGCTAAGAGGCATAAATTGAAGATTACCATTTTGCCTATATTTTTTTTTGTAATATTCACCGGGATTCACACCGTTTTTACCGCCTTTGACAGGAATGACTGTTAGTGAAATGAACTGAGCGAGGGGTGTCGTGTGTGCTGCACCGTGGGGTGTACCGCCACACATGTCTGTACATATGAGTGGCGCAGTTTTTACCGTGTCAGTTTTGTAGACCACATTCCGCTTGTGCAAAGGGGGCCCCCCCGTTCAATGCATGTTTAGCTTTATGTTTAAGAAAAATGGCGTTCCGCTGAGGTGCACTCTGCGGTTACTATTTATGAGCATGCGCTTGTTTATTTGAACGAGGGGACGCTCACCACACTATGCAGTCGTCGCACCTGCCGACTCAGCTTCTTTACCCCCCTAATTGTGTGCCCCCCCCTTTTTTTCCCCTTTCCCCTTAGGGCTAAAGTTTTACACGAGCTGCTCCGGCACAGGGCAGGTAAAATGGGAGCTGCTGGGAGTCCTAACCATTTTGAACAGCCTGATTTTATTGCTAAATGTGAATTACAAGGAAAACATAAACCATTTGAATAACAAGAAGAGCGAAACGAGTGACATAAATGACGATTTGATAAACGTAGATATGAACGAGTTTAGCAACAATGAGAAGGACGAAAGCAGCGATGAAAGTGAAAGAGAGAAAAAATACAACAAAATAAAAATAAGGTACCTATACAGCATTAGCAATTCCAGAGTGTGTTATTACAGCATGTGGATCCTATGCTACTACCTGATCTATTTTTTATGTTTCCTCAGTTTTTTATATGGCATTAGACTTTTCAATAACAATTTAATTAATATATATGCAATCAGGACATGCAAAATTGACAATTTAGCAAATTATATTTTATCCGAAAATACGTTCATAAGCCTTTACTGGGCAATTATTAACTTTAACGTTTTTATGAGCAAGTACACGGACTCGTTTTACATTTCAAATTATATCAAATTGAATTTAGAATTTACCACCGGGAAGAAGAAGCTTTTTTTTTTCGTAAATTATGCTTATCAAATATTGCTACTAAGTTATACCATTTACAAAAATGTGTCTTTATATAATAAGGGTCTATACAATTTGAACCAAATTGTGTGTGCCCTCATCTTTCTCTGTCTAATCCTGTATACTATTTTAGAAATCACCTACGTGTTGGAAATAAATAAGCCCTGCTACTACGGCGTCACGAAGTTGTCCTTCAACTACATTTGGGCCATCATTTACCTATTTGTGATTTTCATTTCCTCCGTCATTTTTTACTTTTCCGTTTTTAGTTATTCCATCAAAGACCAGTTTGTGAATTTTCAAATCATGCTCTGGTTTTTTTTCATTTCCCTAACTTACATAAAAAGGAACCAATTGTTTATAAAGGTTTGA

>Pb_4740|Plasmodium berghei

ATGGCTAAGAGGCATAAATTAAAGATTACTACGTTGTCGATTTTTTTTTTTGTAATATTAACTGGGATTCATACCGTTTTTACCATATTTAACCAGAACGACTGTAAGTTTCATGAAAATATATAATCGTACTTGTACATATAAATAATATGTGATTGCATATGATGCAAATATATGCATGTATTACAAACTGCAAACTTTAGAAAATTGCTGTATCTCATTTTTATCATATGTGCCAATTCCTCTTACAATTATATATATATATATATATATATATATATATATGTATTTATAAATATGTATTAAATGTCCTTTCATGCTATATGCATTTCATTATGCACGGATTGACTCGTTTGTACCATTCCCCATAATTATATATATATACGAACTTTTCATTTCTTTTTGAAGGGATCAAATTTTACACAAGTTGCTCAGGAGAAGGTGATGTAAAATGGGAACTACTATACGTTTTAACCATTTTGAACAGTCTTATCTTAATAATAAATATAAATTACAAAGAAAATATAGACAAACTGAATAATAAGAAGACTGAAATTAGTGATATAAATGATGATTTAATAAATGTTGATATTAACGAGTTTAGTAATAATGAAAGAGATGACAGTGATGATGAACTTGAAAAAGAAAAAAGATACAACAAATTAAAGATAAAAAATCTATATAGTATTAGTAATTCAAGAATTTGCTACTATAGTATGTGGATATTGTGCTATTATTTTATATACTTCTTATGTTTCTTGAGCTTTTTATATGGTATCAGATTATTTAATAACAATTTAATTAATATATATACAATAAGAACATGTAAAATTGAAAAATTAGAAAATTATATAATATCAGAAAATACATTTACAAGTTTATATTGGGTATTTATCAACTTTAACGTATTTATGAGTAAATATACAGACTCCTTTTATGCAATTAATTATGTAAATTTTAATATCGAATTTAGTACAAAAAAGAAAAGAACATTATTTTTCCTAAATTATGCTTATCAGTTATTATTAATAACTTATTCAATATATAAAAATGTATTATTATATAAAAGAGGGTTATATAATTTGAATCAAATTGTATGCTCAATTATTTTCCTTTGTTTAATATTATATACCATTTTCGAAATTACATATGTCTTAGAAATAAATAAGCCATCTTATTACAGTATGCCGAAATTATCATATAATTATGTATGGTCCATTATTTATTTGTTTGTTATTTTTATTTCATCTGTTATATTCTATTTTTCTGTTTACTCATATTCTATAAAAGACACCTTTGTAAATTTTCAAACCATGCTATGGCTTTTTTTCATTTCGTTAACATACATAAAAAGGAAACAATTGTTTATTACGCCGTAA

>Pc_5508|Plasmodium chabaudi

ATGGCTAAGAGGCATAAATTAAAGATTACTACGTTGTCGATTTTTTTTTTTGTAATATTAACCGGGATTCATACCGTTTTTACCACATTTAACCAGAAAGATTGTAAGTTGCATAAAAAAATATAACCTTACTTATACGTATAAATAATATGTGATTATATATGCATGTATTACAACCTGTGAACTTAAAAAAACGATTGTATCTCATTTATATCATATGTACCAATTTCTCTTACAATTATATATATATGAATTTTTTATTTCTTTTTGAAGGGATCAAATTTTACACAAGTTGCTCAGGAGACGGTGATGTAAAATGGGAACTGCTATACGTTTTAACCCTTTTGAACATCTTCATCTTAATGCTAAATGTAAATTACAAAGAAAATATAGACACGCTGAATAATAAGAAGACCGAAATTAGTGATATAAATGATGATTTAATAAATGTTGATATTAACGAGTTTGGTAATAATGAAAGAGATGACAGTGGGGATGAACTTGAAAAAGAAAAAAGATACAACAAATTAAAGATAAAAACTCTATATAGTATTACAAATTCAAGAATTTGCTACTATAGTATGTGGATATTGTGCTATTATTCTATATACTTCTTATGTTTCTTGAGCTTTTTACATGGTATCAGATTATTTAATAACAATTTAATTAACATATATACAATAAGAACATGTAAACTCGAGAAATTAAATAATTATATAATATCAGAAAATACTTTTACAAGTTTGTATTGGGTATTTATCAACTTTAGCGTATTTATGAGCAAGTATACAGACTCTTTTTATGCTATTAATTATGTAAATTTTAATATCGAATTCAGTACAAAAAGAAAAAGAACATTGTTTTTCCTAAACTATGCTTTCCAGTTATTACTAATAACTTATACAATATACAAGAATGTATTATTATATCAAAATGGAGTATATAATTTGAATCAAATTGTATGCTCACTTATCTTTCTTTGTTTAATATTGTATACCATTTTCGAAATCGCATATGTCTTAGAAATAAATAAGCCATCCTATTACAGTATGCCGAAATTACCATATAATTATGTATGGTCCATTATTTATTTATTTGTTATTTTCGTTTCGTCTGTTATATTCTATTTTTCTGTTTATGCATACTCTATAAAAGATACGTTTGTAAATTTTCAAATTATGTTATGGCTTTTTTTCCTTTCGTTAACATACATAAAAAGGAAACAATTATTTATTAAGGCGTAA

>chrPyl_00506|MALPY00506 (PlasmoDB ID: PY01858)

ATGGCTAAAAGGCATAAATTAAAGATTACTACGTTGTCGATTTTTTTTTTTGTAATATTAACTGGGATTCATACCGTTTTTACCACATTTAACCAGAAAGACTGTAAGTTGAATGAAAATATATATAACCGTACTTATACATATAAATAATATGTGATTGCATATGATGCAAATATATGCATGCATTACAAACTGCGAACTTTAGAAAATTATTGTATCTCATTTTTATCATATGTACCAATTCCTCTTACAATTATATATATATGCATTTATAAATATGTATTGAATGCCCTTTTGTGTTATATGCATTGATTGACTCGTTTGTACCTTTCCCTATAATTATATATATATACGAACTTTTCATTTCTTTTTGAAGGGATCAAATTTTACACAAGTTGCTCAGGAGAAGGTGATGTAAAATGGGAACTACTATACGTTTTAACCATTTTGAACAGTCTTCTCTTAATGATAAATATAAATTACAAAGAAAATATAGACAAACTGAATAATAAGAAGACTGAAATTAGTGATATAAATGATGATTTGATAAATGTTGATATTAACGAGTTTGGTAATAATGAAAGAGATGACAGTGGTGATGAACTTGAAAAAGAAAAAAGATACAACAAATTAAAAATAAAAAATTTATATAGCATTAGTAATTCAAGAATTTGCTACTATAGTATGTGGACATTGTGCTATTATTGTATATACTTCTTATGCTTCTTGAGCTTTTTATATGGTATCAGATTATTTAATAACAATTTAATTAATATATATACAATAAGAACATGTAAAATTGAAAAATTAGAAAATTATATAATATCAGAAAATACATTTACAAGTTTATATTGGGTATTTATTAACTTTAACGTATTTATGAGTAAATATACAGACTCCTTTTATGCAATTAATTATGTAAATTTTAATATCGAATTTAGTACAAAAAGGAAAAGAACATTATTTTTCCTAAACTATGCTTATCAATTATTACTAATAACTTATTCAATATATAAAAATGTATTATTATATAAAAAAGGGTTATATAATTTGAATCAAATTGTATGCTCACTTATTTTTCTTTGTTTAATATTATATACCATTTTCGAAATTACATATGTCTTAGAAATAAATAAGCCATCTTATTACAGTATGCCCAAATTATCATATAATTATATATGGTCCATTATTTATTTGTTTGTTATTTTTATTTCATCTGTTATATTCTATTTTTCTGTTTATGCATATTCTATAAAAGACACCTTTGTAAATTTTCAAATCATGTTATGGCTTTTTTTCATTTCATTAACATACATAAAAAGGAAACAATTGTTTATTACGCCGTAA
